# Tracking the Spread of PFAS and EOF in Shanghai Soils over a Decade: Insights from 2007, 2012, and 2017

**DOI:** 10.1021/acs.est.5c10931

**Published:** 2025-12-23

**Authors:** Yao Xiao, Shiyan Chen, Enmiao Jiao, Zhiliang Zhu, Jianfu Zhao, Ying Liu, Leo W. Y. Yeung, Yanling Qiu

**Affiliations:** † Key Laboratory of Yangtze River Water Environment, College of Environmental Science and Engineering, 12476 Tongji University, Shanghai 200092, China; ‡ Shanghai Institute of Pollution Control and Ecological Security, Shanghai 200092, China; § College of Chemistry and Life Sciences, 66344 Zhejiang Normal University, Jinhua 321004, China; ∥ Man-Technology-Environment Research Centre (MTM), School of Science and Technology, Örebro University, Örebro SE-70182, Sweden

**Keywords:** per- and polyfluoroalkyl substances (PFAS), soil, temporal trend, spatial variation, mass balance analysis

## Abstract

Per- and polyfluoroalkyl substances (PFAS) are ubiquitous contaminants in the soil environment, yet their spatiotemporal distribution and long-term trends remain poorly understood. This study investigated the temporal variations and spatial patterns of legacy and emerging PFAS in soil of Shanghai, China, by analyzing 162 soil samples collected in 2007, 2012, and 2017. The results revealed that sum PFAS concentrations ranged from 110 to 18000 pg/g, with an upward trend over the decade (median levels: 1090, 1190, and 2390 pg/g in 2007, 2012, and 2017, respectively). Long-chain PFAS dominated the contaminant profile with perfluoroalkyl carboxylic acids (PFCAs) (1370–2090 pg/g) consistently exceeding perfluoroalkyl sulfonic acids (PFSAs) (145–466 pg/g). For soil samples collected in 2017, a significant amount of F-53B was noted (median: 148 pg/g) as compared to PFOS (median: 95.7 pg/g) and PFOA (median: 1250 pg/g), highlighting the importance of measuring this compound in the Chinese environment. Spatially, PFAS contamination expanded over time, with the pollution center shifting from urban Shanghai to the southwest, forming a new hotspot in that sector. However, no significant correlation was observed between the PFAS distribution and land use. A mass balance analysis indicated that unidentified organofluorine constituted a substantial proportion of extractable organofluorine (90.1, 92.7, and 88.2% in 2007 (target PFAS 11), 2012 (target PFAS 11), and 2017 (target PFAS 20), respectively), highlighting significant gaps in current contaminant identification. These findings provide critical insights into the long-term dynamics of PFAS in a soil environment and underscore the need for future research to prioritize the characterization of unknown fluorine compounds in environmental monitoring.

## Graphical abstract

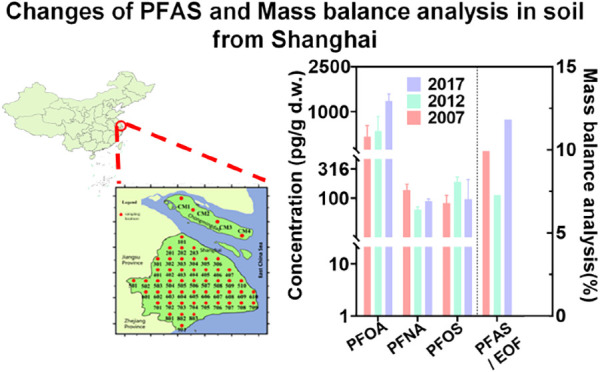

## 1 Introduction

Per- and polyfluoroalkyl substances (PFAS) are a class of anthropogenic fluorinated chemicals, defined as compounds containing at least one fully fluorinated methyl or methylene carbon atom (without any H/Cl/Br/I atom attached to it).^1^ PFAS have been extensively used in industrial and commercial products since the 1950s because of their unique properties, including thermal stability, surface activity, and water- and oil-repellency.^2,3^ While these characteristics explain their widespread use, the frequent detection of PFAS in water,^4–7^ air,^8–10^ soil,^11–14^ and humans^15–17^ is attributable to their environmental persistence, long-range transport potential, and bioaccumulative behavior. Toxicological studies^18–22^ have shown potential adverse effects on human health. These properties and toxic potency ultimately led to perfluorooctanesulfonic acid (PFOS), perfluorooctanoic acid (PFOA), and perfluorohexanesulfonic acid (PFHxS), and their salts and related compounds being regulated under the Stockholm Convention in 2009, 2019, and 2022, respectively.^23^ In May 2025, long-chain perfluorocarboxylic acids (PFCAs) with the molecular formula C*_n_* F_2*n*+1_CO_2_H (where 8 ≤ *n* ≤ 20), their salts, and related compounds were also added to the list (Annex A).^23^ Global restrictions and the elimination of PFAS have therefore shifted the production and application toward emerging PFAS, which are also known as alternatives.^24,25^ Given the ubiquitous occurrence of emerging PFAS in diverse environmental compartments^4,24,26–29^ and their demonstrated toxicity,^25,30,31^ these substitutes are now subject to intensified scientific and regulatory scrutiny.

Soils play a dual role as both important sinks and sources of PFAS in the environment.^32–35^ Pesticide application, atmospheric dry and wet deposition, industrial wastewater and waste discharge, and sewage irrigation are the main pathways for PFAS to enter soils.^34,35^ PFAS in soils can be transported to the atmosphere through volatilization and diffusion, and to surface water and groundwater through leaching and runoff.^36–38^ The presence of PFAS has been investigated globally. PFOS and PFOA are known to be the dominant perfluoroalkyl acids (PFAAs) in soils across 15 countries, with industrial emissions identified as the main source.^39^ The application of aqueous film-forming foams (AFFFs) has also caused significant soil pollution and groundwater contamination.^40^ Another important finding is that the use of biosolids as soil amendments represents a potential vector for PFAS contamination, posing a risk to both soil and groundwater quality.^41,42^ A study on historical land application of biosolids reported soil PFAS concentrations 1–2 orders of magnitude higher than background levels, with quantifiable levels of PFOS and PFOA detected not only in surface soils but also in deep layers and in groundwater at depths of up to 17 m.^42^ Extensive production and application of alternatives have inevitably lead to the release of novel PFAS into soils.^28,34,35,43^ Hexafluoropropylene oxide dimer acid (HFPO-DA, mean concentration: 61.0 pg/g d.w.) has emerged as the predominant novel PFAS, and 3H-perfluoro-3-[(3-methoxy-propoxy)­propanoic acid] (ADONA) has been widely detected (48%) in agricultural soils of North China.^43^ As a consequence of high altitude, wet deposition, and human activity, a variety of novel PFAS [including chlorinated polyfluorinated ether sulfonic acid (Cl-PFESAs), hexafluoropropylene oxide (HFPO) homologs, and fluorotelomer sulfonic acids (FTSAs)] have been identified in Tibetan Plateau soils.^34^ The positive correlations between soil PFAS concentrations and urbanization levels further substantiate anthropogenic activities as primary emission drivers.^44^ Notably, Lan et al. reported a higher detection frequency of 6:2 Cl-PFESA (98%) than PFOS (83%) in soils from the Beijing–Tianjin–Hebei core area, despite comparable concentration ranges (6:2 Cl-PFESA: n.d.–1.20 ng/g; PFOS: n.d.–0.55 ng/g).^28^ This finding underscores the pervasive contamination of novel PFAS in soil environments.

However, existing studies on PFAS contamination in soil environments have primarily provided cross-sectional data. Comprehensive, long-term assessments of PFAS temporal trends remain scarce in complex metropolitan environments with interacting urban sources and land uses. To our knowledge, only Cheng et al.^35^ quantified decadal trends (2011–2021) in agricultural soils of Eastern China, revealing a 28.2% decline in PFOS concentrations, and several studies have reported similar temporal trends of PFAS in soils from other countries.^45–48^ A Korean study showed the seasonal fluctuations in South Korea’s Asan Lake soils, where the highest PFAS levels occurred in summer and the lowest in autumn, with PFOA and PFOS dominating across seasons.^46^ A recent study demonstrated that PFOA and PFOS concentrations in soils decreased within 4 km of the fluorochemical plant from 2010 to 2022, probably due to effective regulatory measures.^48^

Mass balance analysis of fluorine provides a powerful complementary approach to target analysis, enabling comprehensive characterization of fluorinated compound contamination and tracing emission sources of unidentified fluorochemicals.^49,50^ Soil analysis across the Koshi River in Nepal has revealed that target PFAS constituted merely 0 to 1.68% (average: 0.39%) of extractable organofluorine (EOF).^51^ This suggests substantial quantities of unidentified organofluorine remain, underscoring critical gaps in mass balance analysis. Previous study in Liaoning Province, China,^52^ also showed a large proportion of unknown organofluorine.

Among Chinese cities, Shanghai stands out for its high level of industrialization and economic development. Several studies have reported PFAS contamination in Shanghai’s soil environments.^53,54^ However, existing studies have only covered a limited area, leaving significant knowledge gaps regarding temporal trends of PFAS in Shanghai’s soil environments.

The objectives of this study are to (1) characterize the occurrence and composition profiles of PFAS in soils from Shanghai across three time periods (2007, 2012, and 2017); (2) investigate decadal temporal trends and spatial distribution patterns of PFAS in the study area; (3) identify the contamination characteristics of PFAS in different land use types; (4) and perform a mass balance analysis of PFAS in soils to quantify unidentified fluorinated substances.

## 2 Materials and Methods

The analysis of soil samples was performed at two different research laboratories. The samples collected in 2007 and 2012 were analyzed at the University of Toronto (UofT), while those collected in 2017 were analyzed at the MTM Research Centre, Örebro University. The extraction protocol was the same; however, the number of target compounds (13 PFAS for the 2007 and 2012 samples vs 24 PFAS for the 2017 samples) and the instrumental methods were different. They are provided in the Instrumental Analysis section. The lower number of target compounds in the earlier analyses reflects the limited availability of commercial standards at that time; for example, reference materials for HFPO-DA, ADONA, perfluorocyclohexanesulfonic acid (PFECHS), and Cl-PFESAs only became available around 2018–2019, enabling their inclusion in the 2017 analysis.

### 2.1 Chemicals and Reagents

Detailed information about chemicals and reagents is presented in the Supporting Information (SI).

### 2.2 Sampling Sites and Sample Collection

Soil samples (*n* = 54/year) were collected across Shanghai during spring months (March to May) of 2007, 2012, and 2017. Sampling locations are listed in Figure 1. At each site, composite samples were obtained from surface soil (0–10 cm) within a defined area, then freezed-dried, homogenized, sieved (200-mesh), and stored in polypropylene (PP) tubes before analysis. Detailed information about sampling points is provided in the SI.

**Figure 1:**
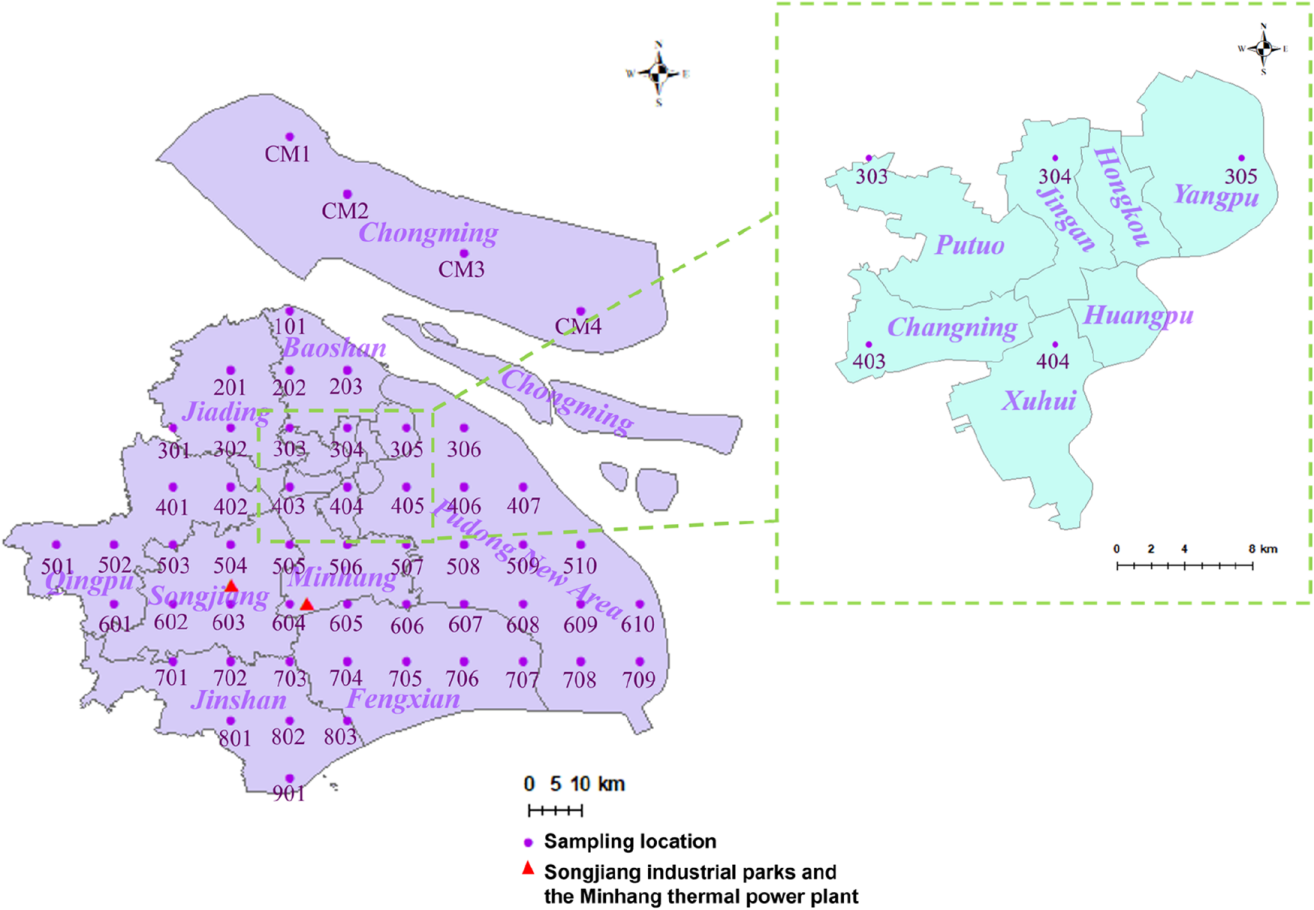
Soil sampling locations in administrative districts of Shanghai.

### 2.3 Extraction and Cleanup

For each sample, two subsamples (about 2.0 g) were weighed. Replicate 1 was spiked with internal standards (IS) for target analysis, replicate 2 without spiking any IS was analyzed for EOF. Samples were extracted using methanol extraction method, combining with a cleanup step using the ion pair method, as described elsewhere.^55^ Detailed information regarding sample extraction and cleanup is elaborated in the SI.

### 2.4 Instrumental Analysis

The majority of target PFAS in this study were analyzed by ultra performance liquid chromatography (UPLC) coupled with a Xevo TQ-S MS/MS in negative mode (ESI^–^) from Waters Corporation, Milford, MA. Detailed instrumental conditions have been reported for MTM^56^ and UofT;^57^ details of the method are provided in the SI.

EOF levels were determined by combustion ion chromatography (CIC). Specific instrument details have been reported elsewhere;^56–58^ the respective details are provided in the SI.

### 2.5 Quality Control and Quality Assurance

Two procedural blanks (ultrapure water) and a mixture of all target analytes were included in each batch. In addition, a matrix blank recovery sample (silica sand) was also analyzed to evaluate the method performance for the 2017 batch samples. Internal standards were added before sample extraction, and RS was added before instrumental analysis to evaluate the recovery of samples for target analysis. Acceptable recovery of matrix spike sample and mass-labeled standards were between 51 and 109%; since some target analytes (e.g., perfluorobutanesulfonic acid (PFBS), perfluorotetradecanoic acid (PFTDA), perfluorohexadecanoic acid (PFHxDA), and perfluorooctadecanoic acid (PFOcDA)) were outside the acceptable range, these results were not reported; details of quality control and quality assurance and the recovery results are provided in the SI and Tables S2 and S3. The limit of quantification was based on the lowest point of the calibration curve that gave at least a signal-to-noise ratio greater than 10, and the theoretical value should be less than 20%. The method detection limit (MDL) was defined as the average of detectable blank level plus 3 times of the standard deviation (Table S2).

For the analysis of EOF, procedural blank samples and quality control (QC) samples were used in each batch. To assess the combustion efficiency of the CIC, a PFOA standard was injected at the beginning of the sample analysis and after every ten samples during instrumental analysis. The instrumental background was by combusting an empty quartz boat, which were combusted between samples to avoid carryover from the previous sample. All detailed information is provided in the SI. Due to no detections in the procedural blanks, the MDL was set as the lowest point of the calibration curve, which was 50 ng of F/mL.

### 2.6 Statistical Analysis

Statistical analyses were performed using IBM SPSS Statistics 25. Differences in PFAS concentrations across the three sampling years were evaluated based on the data’s conformance to parametric assumptions. After testing for normality and homogeneity of variances, a one-way ANOVA was applied to parametric data, while the Kruskal–Wallis H test was used for nonparametric data. Significant results (*p* < 0.05) were followed by post hoc pairwise comparisons (Tukey test for ANOVA; Mann–Whitney U test with Bonferroni correction for Kruskal–Wallis).

## 3 Results and Discussion

### 3.1 Occurrence and Composition Profiles of Target PFAS in Shanghai Soils

The concentration and detection frequencies of the 11 detectable PFAS in 2007 and 2012 and 20 detectable PFAS in 2017 in different soil samples from Shanghai are shown in Table S5. Eleven PFAS, including 8 PFCAs and 3 PFSAs, were ubiquitously detected across all samples.

The sum concentrations of 11 PFAS (∑_11_PFAS) in soils in 2017 were significantly higher than those in 2007 and 2012 (*p* < 0.0167), with ranges of 110–8120 pg/g (mean: 1590 pg/g; median: 1090 pg/g) in 2007, 156–13,300 pg/g (mean: 1640 pg/g; median: 1190 pg/g) in 2012, and 448–18,300 pg/g (mean: 3090 pg/g; median: 2390 pg/g) in 2017 (Figure 2; Table S4). These levels were comparable to those in the east of China (17.6–1950 pg/g),^35^ 31 Chinese provinces (244–13,600 pg/g),^59^ and China’s Fen-Wei Plain (669–3260 pg/g dw).^60^ However, they were observed at lower levels than those found in Shanghai urban soils (141–237 ng/g)^53^ and PFAS-source-adjacent soils in Shanghai (0.64–294 ng/g dw)^54^ and higher than the global background surface-soil levels (<60 pg/g).^61^

**Figure 2:**
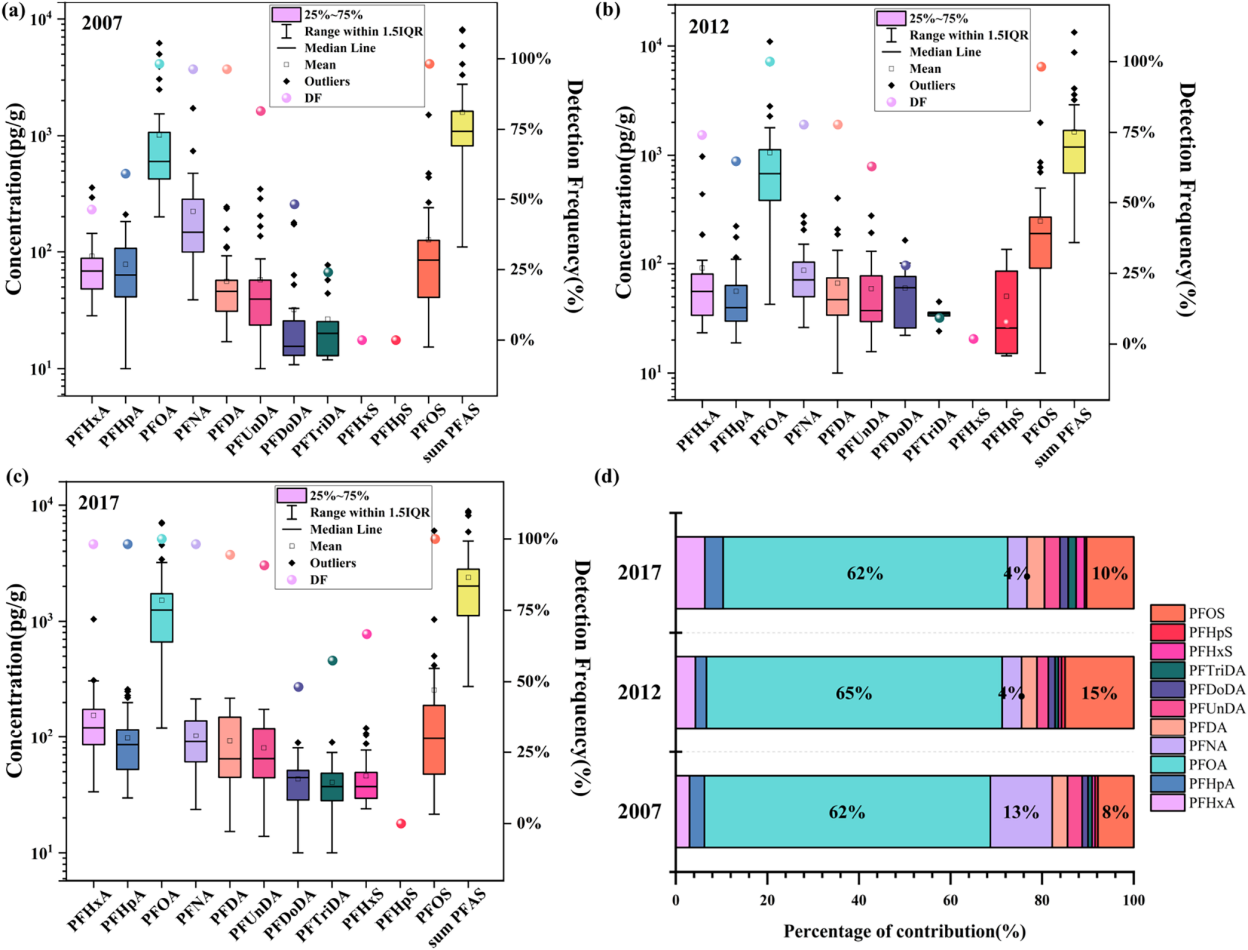
(a–c) Concentrations, detection frequencies, and (d) composition profiles of PFAS in Shanghai soils (The error bars are standard deviation, circle: detection frequency).

Among the 11 PFAS, PFCAs were the dominant species, with mean values of 1450, 1370, and 2090 pg/g, contributing to 91, 84, and 88% of the sum PFAS in 2007, 2012 and 2017, respectively (Figure 2). In contrast, PFSAs levels were 1 order of magnitude lower than those of PFCAs, though the values showed a significant increase in 2012 (270 pg/g) and 2017 (296 pg/g) compared to 2007 (145 pg/g) (*p* < 0.0167) (Table S4 and Figure 2). Furthermore, long-chain PFAS dominated the soil PFAS profile, among which PFOA was the predominant PFAS with the highest detection frequency and concentrations, accounting for 62, 65, and 64% of sum PFAS in 2007, 2012, and 2017, followed by PFOS (7.83, 15.0, and 10.6% from 2007 to 2017, respectively). Elevated PFOA levels have similarly been reported in residential soils from China^59^ and soils near ski resorts in northern China.^62^ The predominance of PFOA in China’s environment stems primarily from intensive historical industrial emissions—accounting for approximately 87% of national releases—particularly in the industrialized central and eastern regions.^38^ This is compounded by effective atmospheric deposition, with an estimated 7.3 t/yr of PFOA adsorbing to particulates and depositing into soils,^63^ alongside persistent sources such as leachate from inadequately contained landfills (accounting for ∼60% of PFOA in waste) and domestic wastewater.^38^ Together, these pathways have led to widespread soil contamination and established PFOA as the dominant PFAS. Notably, the contribution of perfluorononanoic acid (PFNA) was 13.5% in 2007, decreasing to 4.26% in 2012 and 4.25% in 2017.

### 3.2 Temporal Trends of Target PFAS in Soils

The sum PFAS concentrations in Shanghai soils in 2017 were higher than in 2007 and 2012, with median values rising from 1090 pg/g (range: 110–8120 pg/g) in 2007 to 2390 pg/g (range: 448–18300 pg/g) in 2017, and intermediate levels were observed in 2012 (median: 1190 pg/g; range: 156–13300 pg/g) (Table S5). Post hoc pairwise comparisons (Mann–Whitney U test with Bonferroni correction) confirmed that the 2017 concentrations were significantly higher than those in both 2007 and 2012 (*p* < 0.0167), while no significant difference was found between the two earlier time points. Similar temporal variation of PFAS was observed in the Shenzhen Bay area from 1955 to 2020.^64^

#### 3.2.1 For Legacy PFAS

Specifically, PFOA constituted the dominant soil contaminant, with the median concentrations increasing progressively from 599 to 678 pg/g and ultimately increasing significantly to 1250 pg/g (compared with 2007 and 2012, *p* < 0.0167) over the study period. PFOS exhibited a distinct temporal pattern, characterized by a peak in 2012 (median: 189 pg/g) that was significantly higher (Bonferroni-corrected Mann–Whitney U test, *p* < 0.0167) than the levels in both 2007 (median: 82.5 pg/g) and 2017 (median: 95.7 pg/g) (Table S4). Empirical data confirmed the efficacy of global restrictions on PFOS implemented since 2009 and its subsequent listing by the Ministry of Ecology and Environment of the People’s Republic of China (2023 version) with usage limitations,^23,65^ though there are still some exempted uses of PFOS (such as its use for producing firefighting foam agents). Notably, perfluorohexanoic acid (PFHxA) concentration increased sharply by 218% from 2007 to 2017, with significantly elevated levels in 2017 compared to both preceding time points (Bonferroni-corrected Mann–Whitney U test, *p* < 0.0167) (Table S4). Similar upward trends were documented in the North American Great Lakes between 2000 and 2009 and 2015–2019 ^66^ and Laizhou Bay from 2011 to 2018.^67^ In contrast, the concentration of PFNA significantly decreased by 53% over 2007–2017 (*p* < 0.0167). This decline aligned with a 90.5% reduction observed in soils in Eastern China from 2011 to 2021.^35^

#### 3.2.2 For PFAS Alternatives

In terms of emerging PFAS, PFOS alternatives 8:2 Cl-PFESA and 6:2 Cl-PFESA were detected in the majority of soil samples collected in 2017. 6:2 Cl-PFESA (the primary component in commercial chlorinated polyfluoroalkyl ether sulfonates, F-53B) showed the highest mean concentration (469 pg/g), significantly exceeding PFOS levels (Wilcoxon signed-rank test, *p* < 0.05) in this study by 1.86-fold (Table S4). This contrasts with lower 6:2 Cl-PFESA concentrations reported by Cheng et al.^35^ and Li et al.^59^ in agricultural soils of Eastern China (32.7 pg/g) and residential soils of China (156 pg/g dw).

### 3.3 Spatial Analysis of PFAS in Soils

As shown in Figure 3, a distinguishable geographical distribution of ∑PFAS was found in soils of Shanghai in the 10-year period. The decade saw both expanded contaminated areas and elevated concentrations, culminating in pronounced southwestern accumulation. Detailed temporal and spatial variations in PFAS concentrations across Shanghai districts (2007–2017) are presented in Figure S1 and in the SI. Overall, PFCAs remained the predominant PFAS group, with notably higher concentrations observed in the southwestern districts during 2007–2012, followed by a general increase in most regions by 2017. These trends likely reflect regional differences in industrial activity and emission control measures over the past decade. Initial hotspots in 2007 clustered along the shared Songjiang–Minhang district border, where Songjiang Industrial Park and Minhang Thermal Power Plant are located nearby (Figure 3 and Table S6). Observably higher concentrations of PFAS were found near Minhang Thermal Power Plant in 2007 (mean: 8120, 7970, and 5880 pg/g at locations 404, 506, and 605, respectively, Figure 1). Notably, location 506 is close to a fluorine chemical company, where it committed to professional sales of fluorocarbon resin and other fluorine-containing chemical products. Similar hotspots occurred near Songjiang Industrial Park (locations 504 and 505, mean: 1700 and 4100 pg/g, Figure 1), a zone hosting multiple potential PFAS sources including new materials, light industrial machinery, and food packaging. In addition, intensive anthropogenic activity associated with urbanization has led to elevated PFAS concentrations in the central urban area of Shanghai, where remnants of former industrial factories persist. Together, central urban soils showed slightly lower PFAS levels than Songjiang and Minhang, which highlights industrial production as a primary driver governing PFAS occurrence and distribution across the region.

**Figure 3:**
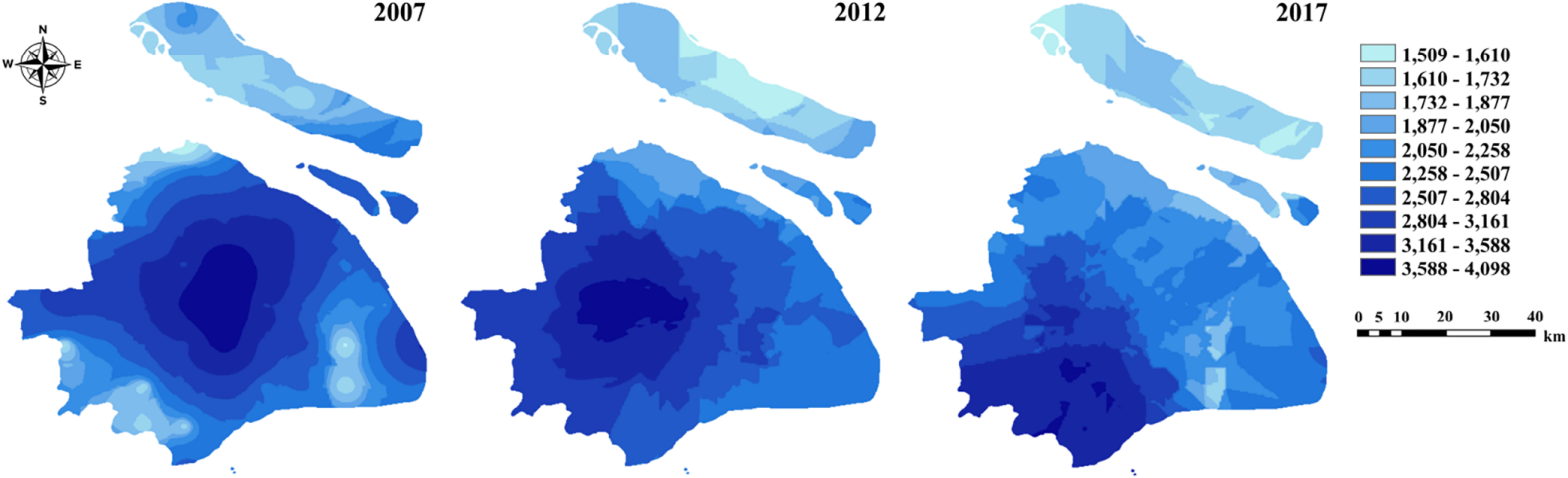
Spatial distribution of sum 11 PFAS concentrations (Σ_11_PFAS) in soils from Shanghai in 2007, 2012, and 2017.

Higher concentrations of PFAS were detected in the soil of Shanghai in 2012 in comparison with those in 2007, with contamination expanding westward and high-concentration zones migrating toward western sectors (Figure 3 and Table S6). This spatial redistribution aligned closely with the distribution pattern of PFAS-related industries and the rapid development of industrial parks in Songjiang and Minhang. These districts prioritized high-tech fluorochemical production, resulting in elevated PFAS emissions to adjacent environments.

By 2017, PFAS contamination had migrated further southwestward, establishing a new pollution center in Jinshan district, an important petrochemical industrial zone in Shanghai (Figure 1 and Figure 3). Stringent fire safety protocols in petrochemical production necessitate aqueous film-forming foam (AFFF) use, elevating PFAS levels in nearby soils. This was exemplified near Shanghai petrochemical (locations 802 and 901, Figure 1), where ∑PFAS mean concentrations reached 18,300 and 5570 pg/g in 2017, respectively (Table S6). Such AFFF-driven contamination was also found at a U.S. Air Force fire-training area, where the soil PFOS concentration reached 2400 μg/kg (median). In addition, the fraction of PFAS was greater in groundwater and solid samples than in AFFF, demonstrating how PFAA precursors transform into PFCAs and PFSAs in the firefighter training area.^68^ Collectively, it is speculated that firefighting operations represented a key contributor to PFAS contamination in Jinshan Chemical Industry Zone soils, especially for PFOS (Figure 1). The dominant PFOA contamination in soils was primarily attributed to industrial sources. Historically, its largest direct emission source has been as a processing aid in fluoropolymer [e.g., polytetrafluoroethylene (PTFE) and polyvinylidene fluoride (PVDF)] manufacturing.^69^ PFOA has been directly used as an additive in various industrial products.^69–71^ Furthermore, PFOA can also be formed indirectly from the degradation of perfluorooctyl sulfonyl (POSF)-based materials or as a byproduct during the chemical processing of fluorotelomer raw materials.^69^

In contrast, despite statistically significant differences between all years (one-way ANOVA, *p* < 0.05), Chongming district maintained consistently low PFAS levels throughout the study period (Figure 1), with concentrations of 705–1090, 505–686, and 1150–1590 pg/g in 2007, 2012, and 2017, respectively (Table S6). A plausible reason may be the proposal of the Chongming Ecological Island in the early 21st century in Shanghai, where industrial phase-outs and a transition to ecological agriculture and tourism likely suppressed contamination sources.

### 3.4 PFAS Distribution and Composition of Shanghai Soils under Different Land Uses

In this study, soils were classified into four functional types based on land-use patterns: abandoned land, agricultural soil, forest land, and green-belt soil. PFAS concentrations varied considerably across land-use types (Figure S2). Forest soils consistently exhibited the highest levels, with ∑PFAS averaging 3930, 5140, and 5000 pg/g in 2007, 2012, and 2017, respectively. Green-belt soils and abandoned soils showed stable values during the period (*p* > 0.05), while the 2017 values of agricultural soils were significantly higher than those in 2007 and 2012 (*p* < 0.0167), with PFOA (240–5000 pg/g) and PFOS (15–1990 pg/g) dominating across years. Notably, Shanghai forest soils contained ∑PFAS concentrations 4–5 orders of magnitude higher than reported for woodland soils in Nepal^51^ and substantially higher than the values observed across mountains in mainland China,^72^ reflecting the severity of local contamination. Despite these clear differences in mean concentrations, statistical analysis revealed no significant correlation between the PFAS distribution and land-use categories. Detailed compound-specific data and extended literature comparisons are provided in the SI and Tables S7–S10.

### 3.5 Extractable Organofluorine (EOF) and Mass Balance Analysis of EOF

An overview of EOF concentrations and mass balance analysis of EOF is presented in Table S11. The mean EOF concentrations in soil collected in 2007, 2012, and 2017 were 23.8 ng F/g (n.d. to 60.2 ng F/g), 20.1 ng F/g (n.d. to 32.8 ng F/g), and 24.2 ng F/g (n.d. to 57.8 ng F/g), respectively. Target PFAS explained 1.68–48.6% (mean: 9.92%) of EOF in 2007 with 11 PFAS, 2.38–32.5% (mean: 7.29%) in 2012 with 11 PFAS, and 0.66–43.6% (mean: 9.27%) with 11 PFAS and 1.10–75.9% (mean: 11.8%) with 20 PFAS in 2017, revealing a significant gap in the fluorine mass balance in soils. In this study, known PFAS accounted for a higher proportion of EOF than reported in soils from 14 estuarine and coastal areas in Liaodong Bay^52^ (0.8%) and soils along the watershed of the Nepali Koshi River^51^ (0.39%). However, observed discrepancies in EOF mass balance analysis between studies must be interpreted with caution since different sample preparation methods may produce slightly different outcomes.

The number of target PFAS measured increased from 11 in both 2007 and 2012 to 20 in 2017. Overall, the 9 newly added PFAS in 2017 contributed an additional 2.55% to the EOF. However, their contribution was much lower than the original 11 PFAS, suggesting that other fluorinated alternatives may have been in use in larger proportions. An observable increase in EOF was observed in 2017 compared to the levels in 2007 and 2012 (*p* > 0.05). A sharply declining value of the contribution of known PFAS to EOF (84.3%) was found in Minhang between 2007 and 2012, followed by Qingpu, Baoshan, Chongming Island, and Pudong New Area (Figure 4a and Table S12). In general, both target PFAS and EOF concentrations in 2017 were not significantly lower than those in 2007 and 2012. However, Jinshan exhibited a sustained upward trajectory, indicating stable organofluorine composition throughout the 2007–2017 period.

**Figure 4:**
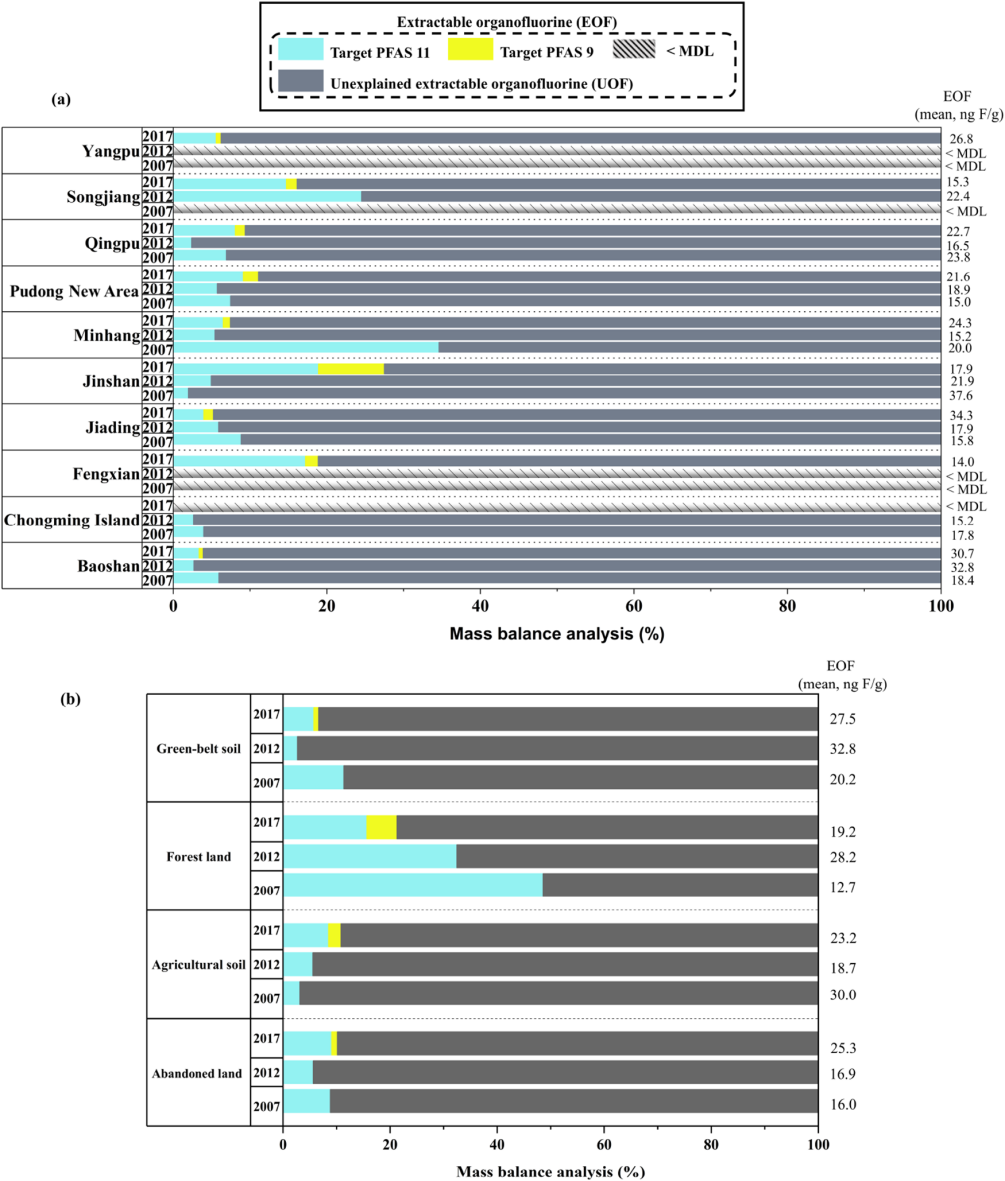
Proportions of PFAS/EOF in various districts (a) and different land uses (b) of Shanghai. The slanted part in the figure was due to the EOF being below the MDL.

Comparable mean EOF concentrations were observed in all land-use types (abandoned, agricultural, forest, and green-belt soils) during the study period (2007–2017) [Table S13 and Figure 4b]. The highest mean PFAS/EOF ratio (48.6%) was recorded in forest soil during 2007, while comparable ratios were observed in the other three soil types. In agricultural soils, the PFAS-to-EOF ratios increased over the study period, with the 2017 value (10.8%) being significantly higher than those in 2007 (3.13%) and 2012 (5.56%) (*p* < 0.0167). Regarding forest soils, target PFAS accounted for mean proportions of 48.6, 32.5, and 21.3% of EOF in 2007, 2012, and 2017, respectively (Figure 4), although these differences were not statistically significant. Overall, the agricultural soil showed a statistically supported increase between 2007 and 2017, whereas forest, green-belt, and abandoned soils exhibited no significant temporal trends.

## 4 Environmental Implications

The decade-long study (2007–2017) reveals critical insights into PFAS contamination dynamics in Shanghai soils. The results demonstrated a significant temporal increase in ΣPFAS concentrations, dominated by PFCAs. Notably, 6:2 Cl-PFESA concentrations were approximately double those of PFOS. Spatial analysis revealed a distinct migration and expansion of PFAS pollution from central Shanghai toward the southwest, ultimately forming a new contamination hotspot in the Jinshan District. This spatial pattern strongly correlates with the distribution of petrochemical industries, highlighting the connection between PFAS contamination and industrial activities. PFAS concentrations also varied significantly by land-use type with forested areas displaying the highest contamination levels. This finding highlights the complex interactions among pollution sources, land-use practices, and environmental transport processes. A limitation of this study is that emerging PFAS could only be included in the 2017 data set, due to the lack of commercially available standards at the time of the 2007 and 2012 analyses. This restricts our ability to assess long-term temporal trends for these newer compounds; however, their inclusion in the 2017 samples still provides valuable information about their occurrence and relative contribution, complementing the temporal patterns observed for legacy PFAS.

Mass balance analysis indicated that known PFAS accounted for only a small fraction of the EOF in soils. This discrepancy suggests that a substantial portion of the EOF consists of unidentified fluorinated compounds that may accumulate alongside the target PFAS over time. The unidentified fraction may comprise various fluorinated compounds, such as fluorinated pesticides (e.g., tefluthrin, fluazinam), which are characterized by typical persistence and high lipophilicity, thereby favoring sequestration by organic carbon in soil.^73^ The substantial proportion of unexplained EOF poses a major challenge for future environmental monitoring and risk assessment efforts. Furthermore, it should be noted that TFA may constitute a non-negligible fraction of PFAS in soil. However, in this study, an additional washing step was implemented during the extraction process to remove interference from the free fluoride. This step markedly reduced the recovery of TFA. As a result, TFA was excluded from the target PFAS analysis. Its contribution may partly explain the proportion of unidentified organofluorine observed in this study.

Given the dynamic nature of PFAS contamination, our research program remains active and evolving. Following our 2023 collection of Shanghai soil samples, current investigations will employ nontarget screening approaches to characterize both known PFAS and unidentified EOF components. Specifically, these efforts will prioritize the identification of fluorinated pesticides and other emerging fluorinated compounds of concern. Future efforts should prioritize methodological advances in extraction and analysis, including the dedicated collection of separate soil aliquots specifically for quantifying ultrashort PFAS such as TFA, to more accurately identify and quantify unknown fluorinated compounds, enabling better source identification, contamination pathway elucidation, and science-based regulatory decision-making.

## Supplementary Material



## Notes

Detailed descriptions of chemicals, sampling points, and analytical method; supporting tables include MRM parameters for PFAS targets, data of quality control and quality assurance, MDL and recovery data in matrix spike recovery; detailed concentrations of PFAS, PFCAs, and PFSAs in Shanghai and its various districts from 2007 to 2017; composition and concentration of PFAS in different land uses of Shanghai; and mass balance analysis in different districts and land uses in Shanghai (PDF)

## ⊥

Zhejiang Environment Technology Co., Ltd., Hangzhou 311100, China
