# Supplementary material for: Tracking the Spread of PFAS and EOF in Shanghai Soils over a Decade: Insights from 2007, 2012, and 2017
Source: Environ Sci Technol. 2025 Dec 23;60(1):1172–81. doi: 10.1021/acs.est.5c10931 (PMC12810246; doi:10.1021/acs.est.5c10931)
Supplement: Supplementary file 1 [file es5c10931_si_001.pdf]

Tracking the Spread of PFAS and EOF in Shanghai Soil Over a Decade: Insights from 2007, 2012, and 2017

Yao Xiao[a,b,1], Shiyan Chen[c,1], Enmiao Jiao[a,b,†], Zhiliang Zhu[a,b], Jianfu Zhao[a,b], Ying Liu[a,b], Leo W. Y. Yeung[d,*], Yanling Qiu[a,b,**]

[a] Key Laboratory of Yangtze River Water Environment, College of Environmental Science and Engineering, Tongji University, Shanghai 200092, China

[b] Shanghai Institute of Pollution Control and Ecological Security, Shanghai 200092, China

[c] College of Chemistry and Life Sciences, Zhejiang Normal University, Jinhua 321004, China

[d] Man-Technology-Environment Research Centre (MTM), School of Science and Technology, Örebro University, SE-70182 Örebro, Sweden

[†] Current address: Zhejiang Environment Technology Co., Ltd., Hangzhou 311100, China

[1] These authors contributed equally to this article.

Corresponding authors: E-mail: Leo.Yeung@oru.se; ylqiu@tongji.edu.cn.

Number of tables: 13

Number figures: 2

Number of pages: 45

Table of content

Text S1. Chemicals and reagents 4

Text S2. Sampling Points..4

Text S3. Sample extraction and clean-up 4

Text S4. Instrumental analysis 5

Text S5. Quality control and quality assurance 5

Text S6. PFCAs and PFSAs Concentrations in Shanghai districts over the past decade (2007-2017) 7

Text S7. PFAS distribution and composition of Shanghai soils under different land uses 8

Table S1. MRM parameter of PFAS for target analysis and internal standard and recovery standards used in PFAS analysis. 10

Table S2. MDL and recovery of native PFAS in matrix spiked samples (silica sand, n=8). ...13

Table S3. Recoveries of internal standards (IS) in QC samples (ultrapure water), matrix blank samples (silica sand) and real samples. 14

Table S4. Concentrations and detection frequencies of PFAS (pg/g dw) in soil samples of Shanghai...15

Table S5. PFAS concentration in the soil from Shanghai (pg/g d.w.)....................................16

Table S6. PFAS concentration in soil from different districts of Shanghai from 2007 to 2017. 19

Table S7. PFAS concentration in abandoned land of Shanghai from 2007 to 2017 (pg/g d.w.). 31

Table S8. PFAS concentration in green-belt soil of Shanghai from 2007 to 2017 (pg/g d.w.). 33

Table S9. PFAS concentration in agricultural soil of Shanghai from 2007 to 2017 (pg/g d.w.). 35

Table S10. PFAS concentration in forest land of Shanghai from 2007 to 2017 (pg/g d.w.). ...37

Table S11. PFAS concentration (expressed in F-equivalent) (ng F/g), EOF concentration (ng F/g), and mass balance analysis of EOF (%) in soil samples from Shanghai..........................39

Table S12. PFAS concentration (expressed in F-equivalent) (ng F/g), EOF concentration (ng F/g), and mass balance analysis of EOF (%) in soil samples from various areas of Shanghai. 40

Table S13. PFAS concentration (expressed in F equivalent) (ng-F/g), EOF concentration (ng F/g), and mass balance analysis of EOF (%) in different soil use from Shanghai. 42

Figure S1. The concentration of PFCAs and PFSAs in soils from Shanghai. 43

Figure S2. The 11 PFAS concentrations (a) and compositional profiles (b) of different land uses in Shanghai..44

References..45

Text S1. Chemicals and reagents

Analytical native standard of PFAS includes eleven native linear perfluorinated carboxylic acids (PFCAs, C_6–C_{16}), five native perfluoroalkane sulfonic acids (PFSAs, C_4, C_6, C_{10} and C_{12} linear; C_8 linear and branched), perfluorooctanesulfonamide, and 6:2 FTSA. The 13 target PFAS for sample collected in 2007 and 2012 include C_6-C_{14} PFCAs, C_4, C_6-C_8 PFSAs, while the 2017 samples include C_6-C_{18} PFCAs, C_4-C_{10} PFSAs, HFPO-DA (also known as GenX), ADONA, perfluorocyclohexane sulfonic acid (PFECHS), chlorinated polyfluoroalkyl ether sulfonic acids (Cl-PFESAs, 6:2 and 8:2). Mass-labelled internal standard (IS) and mass-labelled recovery standard (RS) were purchased from Wellington Laboratories (Guelph, Ontario, Canada) details of these standards are provided in Table S1. Ultrapure water was used throughout the experiments. Methanol (LC-MS grade, ≥ 99.9%), methanol (HPLC grade, ≥ 99.8%), methyl tert-butyl ether (HPLC grade, ≥ 99.8%) were acquired from Sigma-Aldrich (Saint Louis, United States).

Text S2. Sampling Points

At each sampling site, five sub-samples were collected from a 100 m^2 area (0–10 cm depth) using a stainless steel trowel and homogenized to form a composite sample. All samples were freeze-dried for 48 hours in a vacuum freeze-dryer after removing stones, residual roots and other extraneous materials. The freeze-dried soil samples were homogenized using pestle and passed through 200-mesh sieves, then stored in polypropylene (PP) tubes at room temperature before extraction.

The sampling strategy was designed in compliance with *The Technical Specification for soil Environmental monitoring* of China ((HJ/T 166-2004). The entire Shanghai municipality was divided into a grid with ~11 km × 11 km intervals, and sampling sites were positioned at the grid intersections. Chongming Island was incorporated into the design with four evenly distributed sampling points, whereas the smaller Changxing and Hengsha Islands were excluded due to their limited spatial extent.

Text S3. Sample extraction and clean-up

For each sample, two subsamples (about 2.0 g) were weighed into 15 mL PP tubes. Replicate

1 was spiked with internal standards (IS) before extraction and was used for target analysis, replicate 2 was extracted without spiking any IS and was analyzed for EOF. The samples were added 2 mL of alkaline methanol (NaOH in MeOH, 0.2 M), and were vortex-mixed and sonicated for 30 min to allow them to digest. Then 5 mL of MeOH was added and vortexed. The samples were shaken horizontally for 20 min at 250 rpm and then centrifuged for 10 min at 8000 rpm to separate the soil from the liquid phase. The supernatant was transferred to a new 15 mL PP tube. The extraction procedure was repeated with 5 mL of MeOH twice. The MeOH extracts were combined and evaporated to approximately 0.2 mL under gentle stream of nitrogen.

The sample extract was subjected to a cleanup step using the ion pair method. In brief, 2 mL of 0.5 M tetra butyl-ammonium (TBA) solution in water was added to the extract. Then, 5 mL of methyl *tert*-butyl ether (MTBE) was added to the tube. The mixture was shaken horizontally for 15 minutes at 250 rpm and centrifuged for 10 minutes at 8000 rpm to separate the organic and aqueous phases. The top layer (MTBE) was transferred to a new PP tube and the extraction was repeated twice with 3 mL of MTBE. The MTBE extracts were combined and evaporated to approximately 1 mL under a gentle stream of nitrogen.

Text S4. Instrumental analysis

For target PFAS analysis, separation of analytes was performed by a BEH C18 column (2.1 × 100 mm, 1.7 μm at MTM and 2.1 × 50 mm, 1.7 μm at the UofT). The mobile phases were a mixture of MeOH and ultrapure water (30:70, v/v) (A) and MeOH (B), both contained 2 mM ammonium acetate and 5 mM n-methylpiperidine at MTM; whereas at UofT, both phases contained 10 mM ammonium acetate in ultrapure water (A) and methanol (B).

Regarding EOF analysis, the CIC system consists of a combustion module (Analytik Jena, Germany), a 920 Absorber Module, and a 930 Compact IC Flex ion chromatograph module (latter two from Metrohm, Switzerland) at MTM. At UofT, a combination of an automated combustion unit (AQF-100, Mitsubishi Chemical, Japan) and an ion chromatography system (ICS-2100, Dionex Co. Ltd., U.S.) was used.

Text S5. Quality control and quality assurance

Two procedural blanks (ultrapure water) spiked with mass-labelled standards were included in

each batch to monitor possible contamination during extraction and evaluate extraction recovery. In addition, a matrix blank recovery sample (silica sand) spiked with native PFAS and mass-labelled standards prior to extraction (matrix blank spiked sample) was also analyzed to evaluate method performance for the 2017 batch samples. A mixture of all target analytes was injected after every 6 injections to monitor instrumental performance. MeOH injection was performed before and after the standard and sample injection to avoid carry-over. Internal standards were added before sample extraction and RS was added before instrumental analysis to evaluate the recovery of samples for target analysis. As for the concentration of PFAS in replicate 2, IS was added after extraction. The reported EOF concentrations are not recovery corrected. The difference of peak area of target PFAS between replicate 1 (target analysis) and replicate 2 (EOF analysis) were below 20%.

For the analysis of EOF, procedural blank samples were used in each batch to reflect the contamination throughout the process. EOF was quantified using an external calibration curve of PFOA (50-1000 ng F/mL). As for the quality control (QC) sample, a QC sample containing a PFOA solution with an equivalent concentration of 100 ng F/mL (approximately 145 ng/mL) was also extracted in each batch. The resulting recoveries of PFOA were found to be between 69% and 75% for samples from the 2007 and 2012 batches, and between 72% and 83% for samples from the 2017 batch. To assess the combustion efficiency of the CIC, a PFOA standard (approximately 145 ng/mL or 100 ng F/mL) was injected at the beginning of the sample analysis and after every ten samples during instrumental analysis. The combustion efficiency of PFOA was found to be between 81% and 89% for samples from the 2007 and 2012 batches, and between 75% and 86% for samples from the 2017 batch.

Text S6. PFCAs and PFSAs Concentrations in Shanghai districts over the past decade (2007-2017)

Figure S1 illustrated the levels of PFCAs and PFSAs in Shanghai districts over the past decade (2007-2017); PFCAs were shown to be the main group of PFAS. In particular, Minhang (mean: 3680 pg/g) exhibited relatively high PFCAs concentrations in soils compared to other regions in 2007. However, concentration in Minhang declined by 36% between 2007 and 2012. By 2012, Songjiang recorded high PFCAs levels (mean: 2130 pg/g). Subsequently, PFCAs concentrations increased in most regions from 2012 to 2017, with Jinshan experiencing the most pronounced rise (135%). Consequently, in 2017, Songjiang maintained the highest mean PFCAs concentration (3060 pg/g), followed by Jinshan (2800 pg/g) and Minhang (2580 pg/g) (Table S6).

On the other hand, PFSAs trend diverged from those of PFCAs. Relatively higher PFSA concentrations were found in Minhang and Yangpu in 2007, but levels in these regions were comparable or slightly lower by 2012. In Songjiang, PFSAs concentrations increased approximately 4.57-fold between 2007 and 2012, and Baoshan’s mean concentration rose to 318 pg/g in 2012---a 174% increase over the five-year period. In 2017, Jinshan showed highest PFSA concentration (mean: 1380 pg/g), followed by Jiading (436 pg/g). The observed spatial variations in PFAS concentrations are mainly attributed to the distributions of relevant manufacturing industries, leading to elevated release of PFAS into nearby environment.

Text S7. PFAS distribution and composition of Shanghai soils under different land uses

In general, mean sum PFAS concentrations in 2007 followed the order: forest land > green-belt soil > abandoned land > agricultural soil. A similar ranking pattern was observed in 2012, though concentrations in abandoned land decreased relative to 2007 levels. PFAS concentrations in abandon land and agricultural soil in 2017 showed distinctively elevated levels compared to 2012.

In abandoned land, the major contributors were PFOA, PFOS, PFNA, PFHxS, and PFHpA, among which PFOA (1130 and 522 pg/g) and PFOS (78.1 and 190 pg/g) exhibited highest concentrations in 2007 and 2012, respectively, while PFDS (128 pg/g) and 6:2 Cl-PFESA (126 pg/g) emerged with relatively high mean levels in 2017, comparable to PFOS (135 pg/g) in that year (Figure 4 (11 PFAS in all three years) and Table S7 (20 PFAS in all three years)).

Green-belt soil, an essential component of urban spatial functional planning, presented divergent trend from abandoned land. PFAS concentrations in green-belt soil progressively increased over the study period (2000 pg/g, 2250 pg/g, and 2570 pg/g in 2007, 2012, and 2017, respectively). PFOA concentrations remained relatively stable across years (mean: 1390 pg/g in 2007, 1500 pg/g in 2012, and 1540 pg/g in 2017). In contrast, PFOS concentrations in 2017 (120 pg/g) were slightly lower than those in 2007 (132 pg/g) and 2012 (298 pg/g) (Table S8). Due to the scarcity of research on PFAS occurrence in abandoned land and green-belt soil, no further comparative analysis or explanation was pursued.

PFCAs concentrations in agricultural soils remained relatively stable between 2007 and 2012, whereas PFSAs showed slightly elevated levels in 2012 compared to 2007. The mean PFAS concentrations from 2007 to 2017 in our study were 1210, 1340, and 2730 pg/g, respectively, and PFOA (240-5000 pg/g) was the predominant compound accounting for 25.7-85% of total PFAS, followed by PFOS (1.6-62%, 15.3-1990 pg/g) (Table S9), These PFOA and PFOS levels were comparable to those reported in South Korea agricultural soils (PFOA and PFOS were 0.113 and 0.057 ng/g, respectively).[1] Specifically, substantially lower PFAS mean concentration (0.44 ng/g dw) was documented in farmland soils around the Nepali Koshi River,[2] while agricultural areas in Shanghai[3] exhibited markedly higher PFOA (3.28 to 44.0 ng/g) and PFOS (9.20 to 10.4 ng/g) levels.

Notably, the greatest sum PFAS concentration was found in forest land, with average ∑PFAS

values of 3930 pg/g, 5140 pg/g, and 5000 pg/g in 2007, 2012, and 2017, respectively. As expected, PFOA and PFOS were dominant PFAS. PFNA (mean: 858 pg/g) slightly exceeded PFOS (676 pg/g) in 2007. In addition, PFOA reached its maximum concentration (4170 pg/g) in 2012, significantly surpassing PFOS levels (mean: 313 pg/g) (Figure 4(a) and Table S10). The sum PFAS concentrations in our study were significantly higher than in surface forest land samples from 28 mountains across mainland China,[4] and were 4.1-5.4 orders of magnitude higher than the woodland soils around the Nepali Koshi River,[2] reflecting a relatively severe PFAS pollution in forest soil in Shanghai. Furthermore, Tan *et al.* indicated that the mean concentration of PFAS in woodland soils was generally higher than those in farmland soils,[2] which was consistent with the results in the present study. The exact reason for such observation may be related to the frequency of plough. Forest land was covered by dense forests which help soil remain stable and non-ploughed. Nevertheless, agricultural soil is subjected to regular plough and rotation, which could speed up the migration and degradation of PFAS. On the other hand, forest land, with the coverage of thick leaves, has a higher scavenging power of atmospheric pollutants and eventually deposit in soils due to the sufficient organic matter in warm and humid climate.[2] This coincided with findings from Asan Lake, where PFAS concentrations correlated positively with organic carbon (OC) and organic matter (OM) contents.[5] Regarding PFAS alternatives, 6:2 Cl-PFESA (mean: 1300 pg/g) ranked second only to PFOA (2060 pg/g), and was about 2.2 times higher than PFOS.

Table S1. MRM parameter of PFAS for target analysis and internal standard and recovery standards used in PFAS analysis.

Class	Abbreviation	Quantification ion (m/z)	Cone voltage (V)	Collision energy (eV)	Qualification ion (m/z)	Cone voltage (V)	Collision energy (eV)	Internal standard	Recovery standards
PFCAs	PFHxA	312.97/269	20	9	312.97/118.95	20	26	$^{13}C_2$-PFHxA	$^{13}C_5$-PFHxA
	PFHpA	362.97/319	20	10	362.97/168.97	20	16	$^{13}C_4$-PFHpA	$^{13}C_8$-PFOA
	PFOA	412.97/369	20	10	412.97/168.97	20	18	$^{13}C_4$-PFOA	$^{13}C_8$-PFOA
	PFNA	462.99/419	20	12	462.99/219	20	18	$^{13}C_5$-PFNA	$^{13}C_9$-PFNA
	PFDA	512.97/469	20	11	512.97/219	20	18	$^{13}C_2$-PFDA	$^{13}C_6$-PFDA
	PFUnDA	562.97/519	20	12	562.97/268.99	20	18	$^{13}C_2$-PFUnDA	$^{13}C_7$-PFUnDA
	PFDoDA	612.97/569	34	14	612.97/168.96	40	22	$^{13}C_2$-PFDoDA	$^{13}C_7$-PFUnDA
	PFTrDA	662.9/619	20	14	662.9/168.96	20	26	$^{13}C_2$-PFDoDA	$^{13}C_7$-PFUnDA
	PFTDA	712.9/669	20	14	712.9/168.97	20	28	$^{13}C_2$-PFTDA	$^{13}C_7$-PFUnDA
	PFHxDA	812.9/769	30	15	812.9/168.96	42	32	$^{13}C_2$-PFHxDA	$^{13}C_7$-PFUnDA

Class	Abbreviation	Quantification ion (m/z)	Cone voltage (V)	Collision energy (eV)	Qualification ion (m/z)	Cone voltage (V)	Collision energy (eV)	Internal standard	Recovery standards
	PFOcDA	912.9/869	36	15	912.9/168.96	36	36	$^{13}C_2$-PFHxDA	$^{13}C_7$-PFUnDA
PFSAs	PFBS	298.9/98.9	20	26	298.9/79.96	20	26	$^{13}C_3$-PFBS	$^{13}C_3$-PFHxS
	PFPeS	348.9/98.96	20	26	348.9/79.96	20	30	$^{18}O_2$-PFHxS	$^{13}C_3$-PFHxS
	PFHxS	398.9/98.9	20	30	398.9/79.96	20	34	$^{18}O_2$-PFHxS	$^{13}C_3$-PFHxS
	PFHpS	448.97/98.90	20	30	448.97/79.96	20	35	$^{13}C_4$-PFOS	$^{13}C_8$-PFOS
	PFOS	498.97/98.9	20	38	498.97/79.96 (169.03)	20	44 (34)	$^{13}C_4$-PFOS	$^{13}C_8$-PFOS
	PFNS	548.90/98.96	20	38	548.90/79.96	20	44	$^{13}C_4$-PFOS	$^{13}C_8$-PFOS
	PFDS	598.97/98.9	20	42	598.97/79.96	20	58	$^{13}C_4$-PFOS	$^{13}C_8$-PFOS
	PFDoDS	698.9/98.9	20	40	698.9/79.96	20	45	$^{13}C_4$-PFOS	$^{13}C_8$-PFOS
Other PFAS	HFPO-DA	284.92/168.72	20	7	284.92/184.80	20	17	$^{13}C_3$-HFPO-DA	$^{13}C_8$-PFOA
	ADONA	376.97/250.80	15	13	376.97/84.69	15	29	$^{18}O_2$-PFHxS	$^{13}C_3$-PFHxS
	PFECHS	460.84/380.90	2	24	460.84/98.88	2	26	$^{13}C_4$-PFOA	$^{13}C_8$-PFOA
	8:2 Cl-PFESA	630.904/450.98	58	24	630.904/83.027	58	24	$^{13}C_4$-PFOS	$^{13}C_8$-PFOS
	6:2 Cl-PFESA	530.904/350.98	58	24	530.904/83.027	58	24	$^{13}C_4$-PFOS	$^{13}C_8$-PFOS
Internal standard	$^{13}C_2$-PFHxA	314.97/270.0	20	9					
	$^{13}C_4$-PFHpA	366.97/322.0	20	10					

Class	Abbreviation	Quantification ion (m/z)	Cone voltage (V)	Collision energy (eV)	Qualification ion (m/z)	Cone voltage (V)	Collision energy (eV)	Internal standard	Recovery standards
	$^{13}C_4$-PFOA	416.97/327.0	20	10					
	$^{13}C_5$-PFNA	467.99/423.0	20	12					
	$^{13}C_2$-PFDA	514.97/470	20	11					
	$^{13}C_2$-PFUnDA	564.97/520.0	20	12					
	$^{13}C_2$-PFDoDA	614.97/570.0	34	14					
	$^{13}C_2$-PFTDA	714.9/670.0	20	14					
	$^{13}C_2$-PFHxDA	814.9/770.0	30	15					
	$^{13}C_3$-PFBS	301.9/98.9	20	26					
	$^{18}O_2$-PFHxS	402.9/102.9	20	30					
	$^{13}C_4$-PFOS	502.97/98.96	20	38					
	$^{13}C_3$-HFPO-DA	286.9/168.7	20	7					

Table S2. MDL and recovery of native PFAS in matrix spiked samples (silica sand, n=8).

	MDL (pg/g)		matrix spiked recovery	
PFAS	2007 and 2012 samples	2017 samples	2007 and 2012 samples	2017 samples
PFHxA	10	10	86-95%	97-101%
PFHpA	10	10	82-85%	99-105%
PFOA	10	10	86-86%	92-98%
PFNA	10	10	81-86%	95-100%
PFDA	10	10	81-89%	99-100%
PFUnDA	10	10	74-78%	98-102%
PFDoDA	10	10	70-78%	95-99%
PFTrDA	10	10	35-38%	47-105%
PFPeS	NA	10	NA	94-107%
PFHxS	10	10	83-86%	105-115%
PFHpS	10	10	89-92%	109-122%
PFOS	10	10	78-82%	84-104%
PFNS	NA	10	NA	61-79%
PFDS	10	10	78-80%	42-72%
PFDoDS	NA	10	NA	12-89%
HFPO-DA	NA	10	NA	74-88%
ADONA	NA	10	NA	93-114%
PFECHS	NA	10	NA	76-88%
8:2Cl-PFESA	NA	10	NA	32-66%
6:2Cl-PFESA	NA	10	NA	84-91%

Table S3. Recoveries of internal standards (IS) in QC samples (ultrapure water), matrix blank samples (silica sand) and real samples.

	Procedure blank spiked with mass labelled standards		Matrix blank sample spiked recovery	Real sample spiked recovery	
PFAS	2007 and 2012 samples (*n=16*)	2017 samples (*n=8*)	2017 samples (*n=8*)	2007 and 2012 samples (*n=108*)	2017 samples (*n=54*)
$^{13}C_2$-PFHxA	85-92%	92-94%	79-92%	70-91%	65-94%
$^{13}C_4$-PFHpA	81-87%	93-94%	80-91%	63-85%	62-91%
$^{13}C_4$-PFOA	85-89%	91-94%	83-94%	68-93%	63-94%
$^{13}C_5$-PFNA	81-86%	91-92%	82-94%	67-93%	64-93%
$^{13}C_2$-PFDA	83-92%	91-95%	86-93%	66-91%	68-95%
$^{13}C_2$-PFUnDA	75-81%	92-95%	88-95%	59-89%	68-94%
$^{13}C_2$-PFDoDA	71-77%	69-80%	49-92%	60-78%	51-79%
$^{13}C_2$-PFTDA	31-47%	24-53%	13-132%	35-78%	43-126%
$^{13}C_2$-PFHxDA	<20%	4-37%	8-95%	<20%	53-433%
$^{13}C_3$-PFBS	<20%	13-16%	13-17%	<20%	11-21%
$^{18}O_2$-PFHxS	83-88%	91-103%	91-103%	78-86%	77-102%
$^{13}C_4$-PFOS	78-85%	84-94%	90-109%	77-84%	83-100%

Table S4. Concentrations and detection frequencies of PFAS (pg/g dw) in soil samples of Shanghai.

Compounds	2007						2012						2017					
	DF(%)	Min	Max	Mean	SE	Median	DF(%)	Min	Max	Mean	SE	Median	DF(%)	Min	Max	Mean	SE	Median
PFHxA	46.0	10.0	360	47.8	9.46	10.0	74.0	10.0	975	70.2	19.1	38.7	98.0	10.0	1.04 *10^3	153	19.3	120
PFHpA	59.0	10.0	209	51.7	7.00	37.6	65.0	10.0	221	39.8	5.63	29.2	98.0	10.0	259	96.1	8.20	85.2
PFOA	98.0	10.0	6.21 *10^3	993	156	599	100	43.0	1.10 *10^4	1.06 *10^3	231	678	100	120	7.07 *10^3	1.52 *10^3	188	1.25 *10^3
PFNA	96.0	10.0	1.72 *10^3	215	34.2	138	78.0	10.0	275	69.9	7.63	63.8	98.0	10.0	214	101	7.78	89.1
PFDA	96.0	10.0	243	53.4	6.26	43.7	78.0	10.0	398	54.9	8.56	39.9	94.0	10.0	218	89.4	8.55	64.2
PFUnDA	81.0	10.0	350	50.7	8.88	28.2	63.0	10.0	275	41.0	6.66	25.4	91.0	10.0	174	75.1	6.55	62.5
PFDoDA	48.0	10.0	178	20.6	4.38	10.0	28.0	10.0	164	23.9	4.08	10.0	48.0	10.0	89.0	26.7	2.93	10.0
PFTriDA	24.0	10.0	77.1	14.0	12.0	1.64	10.0	9.26	10.0	44.9	12.3	7.40	1.02	10.0	57.0	10.0	89.6	28.1
PFHxS	0	10.0	10.0	10.0	0	10.0	2.00	10.0	51.0	10.8	0.753	10.0	67.0	10.0	120	33.9	3.60	29.5
PFHpS	0	10.0	10.0	10.0	0	10.0	7.00	10.0	135	13.0	2.36	10.0	0	10.0	10.0	10.0	0	10.0
PFOS	98.0	10.0	1.51 *10^3	125	29.0	82.5	98.0	10.0	1.99 *10^3	246	40.9	189	100	10.0	6.02 *10^3	252	111	95.7
6:2 Cl-PFESA	N/A	N/A	N/A	N/A	N/A	N/A	N/A	N/A	N/A	N/A	N/A	N/A	93.0	10.0	8.67*10^3	469	184	148
$\sum_8$PFCAs		80.0	7.51 *10^3	1.45 *10^3	196	961		113	1.24 *10^4	1.37 *10^3	262	930		190	8.46 *10^3	2.09 *10^3	221	1.79 *10^3
$\sum_3$PFSAs		30.0	1.53 *10^3	145	29.0	102		30.0	2.01 *10^3	270	41.1	209		42.0	6.10 *10^3	296	112	125

Compounds	2007						2012						2017					
	DF(%)	Min	Max	Mean	SE	Median	DF(%)	Min	Max	Mean	SE	Median	DF(%)	Min	Max	Mean	SE	Median
$\sum_{11}$PFAS		110	8.12 *10^3	1.59 *10^3	217	1.09 *10^3		156	1.33 *10^4	1.64 *10^3	282	1.19 *10^3		275	8.87 *10^3	2.38 *10^3	259	2.01 *10^3

DF: detection frequency; SE: standard error; N/A: not analyzed for samples in 2007 and 2012.

Table S5. PFAS concentration in the soil from Shanghai (pg/g d.w.).

Compounds	2007						2012						2017					
	DF%	Min	Max	Mean	SE	Median	DF%	Min	Max	Mean	SE	Median	DF%	Min	Max	Mean	SE	Median
PFCAs																		
PFHxA	46.0	10.0	360	47.8	9.46	10.0	74.0	10.0	975	70.2	19.1	38.7	98.0	10.0	1.04*10 ^3	153	19.3	120
PFHpA	59.0	10.0	209	51.7	7.00	37.6	65.0	10.0	221	39.8	5.63	29.2	98.0	10.0	259	96.1	8.20	85.2
PFOA	98.0	10.0	6.21*10 ^3	993	156	599	100	43.0	1.10*10 ^4	1.06*10 ^3	231	678	100	120	7.07*10 ^3	1.52* 10^3	188	1.25*10 ^3
PFNA	96.0	10.0	1.72*10 ^3	215	34.2	138	78.0	10.0	275	69.9	7.63	63.8	98.0	10.0	214	101	7.78	89.1
PFDA	96.0	10.0	243	53.4	6.26	43.7	78.0	10.0	398	54.9	8.56	39.9	94.0	10.0	218	89.4	8.55	64.2
PFUnDA	81.0	10.0	350	50.7	8.88	28.2	63.0	10.0	275	41.0	6.66	25.4	91.0	10.0	174	75.1	6.55	62.5
PFDoDA	48.0	10.0	178	20.6	4.38	10.0	28.0	10.0	164	23.9	4.08	10.0	48.0	10.0	89.0	26.7	2.93	10.0
PFTrDA	24.0	10.0	77.1	14.0	12.0	1.64	10.0	9.26	10.0	44.9	12.3	7.40	1.02	10.0	57.0	10.0	89.6	28.1
PFSAs																		
PFPeS	N/A	N/A	N/A	N/A	N/A	N/A	N/A	N/A	N/A	N/A	N/A	N/A	0	10.0	10.0	10.0	0	10.0
PFHxS	0	10.0	10.0	10.0	0	10.0	2.00	10.0	51.0	10.8	0.753	10.0	67.0	10.0	120	33.9	3.60	29.5
PFHpS	0	10.0	10.0	10.0	0	10.0	7.00	10.0	135	13.0	2.36	10.0	0	10.0	10.0	10.0	0	10.0

Compounds	2007						2012						2017					
	DF%	Min	Max	Mean	SE	Median	DF%	Min	Max	Mean	SE	Median	DF%	Min	Max	Mean	SE	Median
PFOS	98.0	10.0	1.51*10^3	125	29.0	82.5	98.0	10.0	1.99*10^3	246	40.9	189	100	10.0	6.02*10^3	252	111	95.7
PFNS	N/A	N/A	N/A	N/A	N/A	N/A	N/A	N/A	N/A	N/A	N/A	N/A	0	10.0	10.0	10.0	0	10.0
PFDS	N/A	N/A	N/A	N/A	N/A	N/A	N/A	N/A	N/A	N/A	N/A	N/A	100	40.3	313	132	7.48	124
PFDoDS	N/A	N/A	N/A	N/A	N/A	N/A	N/A	N/A	N/A	N/A	N/A	N/A	17.0	10.0	110	17.6	2.62	10.0
Other PFAS																		
HFPO-DA	N/A	N/A	N/A	N/A	N/A	N/A	N/A	N/A	N/A	N/A	N/A	N/A	0	10.0	10.0	10.0	0	10.0
ADONA	N/A	N/A	N/A	N/A	N/A	N/A	N/A	N/A	N/A	N/A	N/A	N/A	0	10.0	10.0	10.0	0	10.0
PFECHS	N/A	N/A	N/A	N/A	N/A	N/A	N/A	N/A	N/A	N/A	N/A	N/A	0	10.0	10.0	10.0	0	10.0
8:2 Cl-PFESA	N/A	N/A	N/A	N/A	N/A	N/A	N/A	N/A	N/A	N/A	N/A	N/A	54.0	10.0	469	41.5	10.6	12.1
6:2 Cl-PFESA	N/A	N/A	N/A	N/A	N/A	N/A	N/A	N/A	N/A	N/A	N/A	N/A	93.0	10.0	8.67*10^3	469	184	148
$\sum_{8}$PFCAs		80.0	7.51*10^3	1.45*10^3	196	961		113	1.24*10^4	1.37*10^3	262	930		190	8.46*10^3	2.09*10^3	221	1.79*10^3
$\sum_{3}$PFSAs		30.0	1.53*10^3	145	29.0	102		30.0	2.01*10^3	270	41.1	209		42.0	6.10*10^3	296	112	125
$\sum_{11}$PFAS		110	8.12*10^3	1.59*10^3	217	1.09*10^3		156	1.33*10^4	1.64*10^3	282	1.19*10^3		275	8.87*10^3	2.38*10^3	259	2.01*10^3
$\sum$PFCAs		80.0	7.51*10^3	1.45*10^3	196	961		113	1.24*10^4	1.37*10^3	262	930		190	8.46*10^3	2.09*10^3	221	1.79*10^3
$\sum$PFSAs		30.0	1.53*10^3	145	29.0	102		30.0	2.01*10^3	270	41.1	209		175	6.33*10^3	466	113	321

Compounds	2007						2012						2017					
	DF%	Min	Max	Mean	SE	Median	DF%	Min	Max	Mean	SE	Median	DF%	Min	Max	Mean	SE	Median
∑PFAS		110	8.12*10^3	1.59*10^3	217	1.09*10^3		156	1.33*10^4	1.64*10^3	282	1.19*10^3		448	1.83*10^4	3.09*10^3	381	2.39*10^3

N/A: not analyzed for samples in 2007 and 2012.

Table S6. PFAS concentration in soil from different districts of Shanghai from 2007 to 2017.

		Concentration (pg/g d.w)	PFHxA	PFHpA	PFOA	PFNA	PFDA	PFUnDA	PFDoDA	PFTriDA
Baoshan (n=2)	2007	**Mean**	10.0	67.3	809	187	41.5	31.5	12.9	10
		**SD**	0	27.3	206	59.9	3.37	7.66	0.999	0
		**Median**	10.0	67.3	809	187	41.5	31.5	12.9	10
	2012	**Mean**	35.3	36.6	412	18.0	29.3	10.0	10	10
		**SD**	25.3	26.6	167	8.05	19.3	0	0	0
		**Median**	35.3	36.6	412	18.0	29.3	10.0	10	10
	2017	**Mean**	93.5	82.1	1.01*10^3	91.7	57.1	58.2	10	21.7
		**SD**	24.1	32.2	351	24.7	20.0	20.8	0	11.7
		**Median**	93.5	82.1	1.01*10^3	91.7	57.1	58.2	10	21.7
Chongming Island (n=4)	2007	**Mean**	10.0	34.8	506	206	36.7	34.0	10.0	10.0
		**SD**	0	24.8	120	135	7.76	7.61	0	0
		**Median**	10.0	33.8	496	179	33.8	34.1	10.0	10.0
	2012	**Mean**	24.7	16.2	364	63.3	17.3	14.0	23.7	10.0
		**SD**	8.90	10.7	75.6	17.0	7.66	6.87	23.7	0
		**Median**	27.6	10.0	385	56.6	15.7	10.0	10.0	10.0
	2017	**Mean**	83.9	54.7	597	77.3	44.9	60.7	10.0	23.9
		**SD**	27.7	12.1	101	15.1	10.4	13.9	0	8.36
		**Median**	87.0	51.9	622	77.5	44.9	65.8	10.0	26.8
Fengxian	2007	**Mean**	43.3	34.0	650	129	35.6	24.6	10.9	10.0

		Concentration (pg/g d.w)	PFHxA	PFHpA	PFOA	PFNA	PFDA	PFUnDA	PFDoDA	PFTriDA
(n=4)		**SD**	23.1	28.8	316	43.8	11.8	15.3	1.63	0
		**Median**	44.7	22.7	627	126	34.0	20.2	10.0	10.0
	2012	**Mean**	48.5	21.2	754	79.5	44.1	31.2	26.4	10.0
		**SD**	27.4	19.4	286	28.1	21.9	13.9	28.4	0
		**Median**	34.8	10.0	769	80.0	48.2	33.3	10.0	10.0
	2017	**Mean**	182	95.3	1.29*10^3	76.8	49.0	44.2	13.1	14.2
		**SD**	82.5	64.6	768	24.0	9.51	14.5	5.38	7.23
		**Median**	170	71.9	964	69.6	49.9	42.6	10.0	10.0
Jiading (n=6)	2007	**Mean**	37.7	67.6	533	141	30.9	23.8	12.0	10.9
		**SD**	31.0	63.3	448	101	14.5	15.5	3.30	1.28
		**Median**	24.8	43.2	402	120	30.8	17.9	10.0	10.0
	2012	**Mean**	23.3	33.9	579	39.9	55.3	26.5	22.0	15.8
		**SD**	13.5	23.7	396	42.5	59.5	26.3	26.8	13.0
		**Median**	21.4	32.0	459	22.9	32.8	15.1	10.0	10.0
	2017	**Mean**	81.9	72.4	948	81.7	74.4	58.1	27.1	26.4
		**SD**	42.8	52.5	914	63.6	53.5	42.8	19.7	19.9
		**Median**	72.1	53.1	592	56.1	58.2	45.9	22.9	17.4
Jinshan (n=7)	2007	**Mean**	12.6	30.0	454	195	39.2	83.1	16.5	12.5
		**SD**	6.43	34.8	153	111	30.1	113	8.60	4.06
		**Median**	10.0	10.0	390	170	22.0	51.4	10.0	10.0
	2012	**Mean**	46.7	36.9	850	107	75.5	55.5	11.7	10.0
		**SD**	21.0	21.2	228	60.3	55.2	57.5	4.24	0
		**Median**	51.8	26.8	871	83.1	60.2	37.3	10.0	10.0

		Concentration (pg/g d.w)	PFHxA	PFHpA	PFOA	PFNA	PFDA	PFUnDA	PFDoDA	PFTriDA
	2017	**Mean**	173	120	1.93*10^3	184	167	139	41.4	44.0
		**SD**	39.3	57.8	566	23.3	27.4	20.3	7.16	5.14
		**Median**	170	97.9	1.89*10^3	193	176	136	44.8	44.4
Minhang (n=6)	2007	**Mean**	74.7	95.8	2.80*10^3	420	103	99.7	55.3	31.6
		**SD**	128	87.2	2.34*10^3	590	77.9	107	58.5	26.4
		**Median**	10.0	82.3	2.28*10^3	204	78.2	43.7	35.4	17.7
	2012	**Mean**	109	61.3	2.03*10^3	56.7	43.2	36.4	21.0	13.9
		**SD**	148	60.0	2.27*10^3	35.6	23.2	27.4	18.5	8.67
		**Median**	47.5	28.1	857	67.7	42.3	26.3	10.0	10.0
	2017	**Mean**	100	95.4	2.14*10^3	82.2	65.0	53.8	25.8	19.9
		**SD**	75.5	86.8	2.45*10^3	67.8	53.0	45.9	18.7	16.7
		**Median**	67.8	51.6	840	62.2	50.5	41.3	17.6	10.0
Pudong New Area (n=13)	2007	**Mean**	64.6	37.8	843	201	56.1	37.2	24.5	14.0
		**SD**	60.3	30.3	557	195	53.3	39.5	43.0	9.25
		**Median**	51.8	31.3	582	112	48.4	23.6	10.0	10.0

		Concentration (pg/g d.w)	PFHxA	PFHpA	PFOA	PFNA	PFDA	PFUnDA	PFDoDA	PFTriDA
	2012	**Mean**	64.3	35.7	790	57.0	36.4	25.8	24.3	10.0
		**SD**	45.6	27.5	439	39.2	22.6	13.9	26.0	0
		**Median**	57.3	33.3	858	64.1	39.9	29.6	10.0	10.0
	2017	**Mean**	240	83.2	1.48*10^3	67.3	56.1	43.7	17.0	14.8
		**SD**	244	49.2	1.64*10^3	26.7	43.4	20.7	21.0	9.30
		**Median**	167	66.6	1.10*10^3	67.8	53.2	47.3	10.0	10.0
Qingpu (n=6)	2007	**Mean**	90.4	38.7	961	205	59.6	45.7	12.8	10.0
		**SD**	93.7	29.0	443	138	28.5	25.7	2.35	0
		**Median**	60.4	30.5	968	166	52.3	43.5	12.7	10.0
	2012	**Mean**	29.1	30.4	468	48.7	44.4	48.1	13.3	10.0
		**SD**	27.9	22.4	329	30.4	32.8	46.7	7.36	0
		**Median**	10.0	23.5	307	54.4	35.3	22.2	10.0	10.0
	2017	**Mean**	110	122	1.35*10^3	122	141	115	43.7	44.4
		**SD**	19.0	42.0	386	25.5	46.0	32.0	20.5	14.3
		**Median**	101	122	1.41*10^3	131	140	112	44.5	39.5
Songjiang (n=5)	2007	**Mean**	38.1	70.3	1.22*10^3	241	59.1	61.7	15.8	12.4
		**SD**	44.7	42.9	956	131	26.5	38.8	5.34	3.89
		**Median**	10.0	63.3	763	316	46.8	45.5	13.0	10.0

	Concentration (pg/g d.w)	PFHxA	PFHpA	PFOA	PFNA	PFDA	PFUnDA	PFDoDA	PFTriDA
2012	**Mean**	253	84.3	3.35*10^3	153	154	117	64.4	20.1
	**SD**	362	76.1	3.92*10^3	78.7	128	87.6	56.6	12.4
	**Median**	86.5	50.8	1.79*10^3	126	121	101	61.4	10.0
2017	**Mean**	152	131	2.29*10^3	139	139	110	45.7	51.1
	**SD**	57.7	61.7	1.14*10^3	58.9	69.9	54.5	21.9	27.9
	**Median**	150	121	1.73*10^3	157	174	143	48.5	61.8
Yangpu (n=1) 2007	**Mean**	10.0	115	1.43*10^3	93.7	56.2	52.6	10.0	10.0
2012	**Mean**	33.5	28.6	502	42.2	33.9	39.9	10.0	24.2
2017	**Mean**	149	113	1.47*10^3	87.2	63.9	61.5	10.0	21.9

Table S6. PFAS concentration in soil from different districts of Shanghai from 2007 to 2017.(Continue table).

		Concentration (pg/g d.w)	PFPeS	PFHxS	PFHpS	PFOS	PFNS	PFDS	PFDoDS	HFPO-DA	ADONA	PFECHS	8:2 Cl-PFESA	6:2 Cl-PFESA	∑PFCAs	∑PFSAs	∑PFAS
Baoshan (n=2)	2007	**Mean**	N/A	10.0	10.0	96.3	N/A	N/A	N/A	N/A	N/A	N/A	N/A	N/A	1.17*10^3	116	1.28*10^3
		**SD**		0	0	17.5									281	17.5	299
		**Median**		10.0	10.0	96.3									1.17*10^3	116	1.28*10^3
	2012	**Mean**	N/A	10.0	10.0	298	N/A	N/A	N/A	N/A	N/A	N/A	N/A	N/A	561	318	880
		**SD**		0	0	202									246	202	448
		**Median**		10.0	10.0	298									561	318	880
	2017	**Mean**	10.0	10.0	10.0	74.1	10.0	64.3	10.0	10.0	10.0	10.0	11.9	141	1.42*10^3	188	1.79*10^3
		**SD**	0	0	0	7.36	0	2.71	0	0	0	0	1.90	15.3	484	10.1	481
		**Median**	10.0	10.0	10.0	74.1	10.0	64.3	10.0	10.0	10.0	10.0	11.9	141	1.42*10^3	188	1.79*10^3
Chongming Island (n=4)	2007	**Mean**	N/A	10.0	10.0	53.3	N/A	N/A	N/A	N/A	N/A	N/A	N/A	N/A	847	73.3	921
		**SD**		0	0	24.5									127	24.5	141
		**Median**		10.0	10.0	46.2									887	66.2	945
	2012	**Mean**	N/A	10.0	10.0	63.6	N/A	N/A	N/A	N/A	N/A	N/A	N/A	N/A	533	83.6	616
		**SD**		0	0	16.6									80.3	16.6	73.6
		**Median**		10.0	10.0	64.6									561	84.6	637
	2017	**Mean**	10.0	10.0	10.0	47.6	10.0	209	52.6	10.0	10.0	10.0	10.1	76.1	952	349	1.42*10^3
		**SD**	0	0	0	12.6	0	13.3	4.86	0	0	0	0.209	17.9	160	22.0	167

		Concentration (pg/g d.w)	PFPeS	PFHxS	PFHpS	PFOS	PFNS	PFDS	PFDoDS	HFPO-DA	ADONA	PFECHS	8:2 Cl-PFESA	6:2 Cl-PFESA	∑PFCAs	∑PFSAs	∑PFAS
		**Median**	10.0	10.0	10.0	46.9	10.0	208	51.3	10.0	10.0	10.0	10.0	74.6	1.01*10^3	345	1.46*10^3
Fengxian (n=4)	2007	**Mean**	N/A	10.0	10.0	67.6	N/A	N/A	N/A	N/A	N/A	N/A	N/A	N/A	938	87.6	1.03*10^3
		**SD**		0	0	37.2									404	37.2	438
		**Median**		10.0	10.0	63.9	N/A	N/A	N/A	N/A	N/A	N/A	N/A	N/A	875	83.9	968
	2012	**Mean**	N/A	10.0	10.0	153									1.02*10^3	173	1.19*10^3
		**SD**		0	0	63.0									380	63.0	438
		**Median**		10.0	10.0	131									1.04*10^3	151	1.19*10^3
	2017	**Mean**	10.0	36.9	10.0	51.1	10	155	10.0	10.0	10.0	10.0	13.3	196	1.77*10^3	283	2.29*10^3
		**SD**	0	1.98	0	13.9	0	18.3	0	0	0	0	2.73	79.9	956	10.9	942
		**Median**	10.0	36.0	10.0	53.3	10	156	10.0	10.0	10.0	10.0	12.9	169	1.36*10^3	282	1.90*10^3
Jiading (n=6)	2007	**Mean**	N/A	10.0	10.0	50.5	N/A	N/A	N/A	N/A	N/A	N/A	N/A	N/A	857	70.5	928
		**SD**		0	0	33.4									583	33.4	614
		**Median**		10.0	10.0	41.7									765	61.7	826
	2012	**Mean**	N/A	10.0	10.0	143	N/A	N/A	N/A	N/A	N/A	N/A	N/A	N/A	796	163	959
		**SD**		0	0	65.6									575	65.6	595
		**Median**		10.0	10.0	154									587	174	711

	Concentration (pg/g d.w)		PFPeS	PFHxS	PFHpS	PFOS	PFNS	PFDS	PFDoDS	HFPO-DA	ADONA	PFECHS	8:2 Cl-PFESA	6:2 Cl-PFESA	∑PFCAs	∑PFSAs	∑PFAS
	2017	**Mean**	10.0	13.8	10.0	291	10.0	91.4	10.0	10.0	10.0	10.0	102	497	1.37*10^3	436	2.43*10^3
		**SD**	0	8.44	0	360	0	14.6	0	0	0	0	89.5	553	1.20*10^3	354	1.60*10^3
		**Median**	10.0	10.0	10.0	112	10.0	91.1	10.0	10.0	10.0	10.0	66.5	314	900	247	2.03*10^3
Jinshan (n=7)	2007	**Mean**	N/A	10.0	10.0	106	N/A	N/A	N/A	N/A	N/A	N/A	N/A	N/A	843	126	969
		**SD**		0	0	63.8									251	63.8	305
		**Median**		10.0	10.0	125									904	145	1.07*10^3
	2012	**Mean**	N/A	10.0	10.8	225	N/A	N/A	N/A	N/A	N/A	N/A	N/A	N/A	1.19*10^3	246	1.44*10^3
		**SD**		0	2.05	59.8									316	60.7	296
		**Median**		10.0	10.0	254									1.26*10^3	274	1.44*10^3
	2017	**Mean**	10.0	74.4	10.0	1.05*10^3	10.0	193	36.4	10.0	10.0	10.0	95.7	1.63*10^3	2.80*10^3	1.38*10^3	5.94*10^3
		**SD**	0	22.6	0	2.03*10^3	0	51.7	33.2	0	0	0	153	2.88*10^3	627	2.02*10^3	5.09*10^3
		**Median**	10.0	74.5	10.0	218	10.0	170	26.6	10.0	10.0	10.0	31.7	532	2.74*10^3	577	3.76*10^3
Minh	2007	**Mean**	N/A	10.0	10.0	384	N/A	N/A	N/A	N/A	N/A	N/A	N/A	N/A	3.68*10	404	4.08*10

		Concentration (pg/g d.w)	PFPeS	PFHxS	PFHpS	PFOS	PFNS	PFDS	PFDoDS	HFPO-DA	ADONA	PFECHS	8:2 Cl-PFESA	6:2 Cl-PFESA	∑PFCAs	∑PFSAs	∑PFAS
ang															^3		^3
(n=6)		**SD**		0	0	525									2.95*10^3	525	3.33*10^3
		**Median**		10.0	10.0	144									3.27*10^3	164	3.43*10^3
		**Mean**	N/A	10.0	14.3	280	N/A	N/A	N/A	N/A	N/A	N/A	N/A	N/A	2.37*10^3	304	2.67*10^3
	2012	**SD**		0	9.58	233									2.57*10^3	242	2.80*10^3
		**Median**		10.0	10.0	223									1.06*10^3	243	1.31*10^3
		**Mean**	10.0	30.6	10.0	167	10.0	125	10.0	10.0	10.0	10.0	47.5	113	2.58*10^3	363	3.14*10^3
	2017	**SD**	0	11.8	0	167	0	42.0	0	0	0	0	54.3	93.4	2.74*10^3	164	2.91*10^3
		**Median**	10.0	30.0	10.0	118	10.0	123	10.0	10.0	10.0	10.0	10.0	110	1.14*10^3	333	1.64*10^3
Pudong New Area (n=13	2007	**Mean**	N/A	10.0	10.0	104	N/A	N/A	N/A	N/A	N/A	N/A	N/A	N/A	1.28*10^3	124	1.40*10^3
		**SD**		0	0	115									773	115	823
		**Median**		10.0	10.0	84.9									939	105	996
	2012	**Mean**	N/A	13.1	20.0	269	N/A	N/A	N/A	N/A	N/A	N/A	N/A	N/A	1.04*10	302	1.35*10

		Concentration (pg/g d.w)	PFPeS	PFHxS	PFHpS	PFOS	PFNS	PFDS	PFDoDS	HFPO-DA	ADONA	PFECHS	8:2 Cl-PFESA	6:2 Cl-PFESA	∑PFCAs	∑PFSAs	∑PFAS
)															^3		^3
		**SD**		10.8	33.3	504									532	505	812
		**Median**		10.0	10.0	150									1.20*10^3	170	1.62*10^3
	2017	**Mean**	10.0	26.6	10.0	70.6	10.0	125	14.0	10.0	10.0	10.0	24.9	488	2.00*10^3	266	2.81*10^3
		**SD**	0	12.3	0	29.9	0	42.1	14.0	0	0	0	48.1	1.38*10^3	1.95*10^3	52.0	2.32*10^3
		**Median**	10.0	27.5	10.0	81.6	10.0	115	10.0	10.0	10.0	10.0	10.0	93.1	1.54*10^3	269	2.22*10^3
Qingpu (n=6)	2007	**Mean**	N/A	10.0	10.0	107	N/A	N/A	N/A	N/A	N/A	N/A	N/A	N/A	1.42*10^3	127	1.55*10^3
		**SD**		0	0	56.6									574	56.6	602
		**Median**		10.0	10.0	98.2									1.45*10^3	118	1.57*10^3
	2012	**Mean**	N/A	10.0	10.0	269	N/A	N/A	N/A	N/A	N/A	N/A	N/A	N/A	692	289	981
		**SD**		0	0	158									410	158	454
		**Median**		10.0	10.0	283									604	303	959
	2017	**Mean**	10.0	38.9	10.0	169	10.0	97.0	10.0	10.0	10.0	10.0	18.5	266	2.05*10^3	344	2.70*10^3
		**SD**	0	24.4	0	106	0	33.2	0	0	0	0	12.5	156	494	110	658
		**Median**	10.0	32.9	10.0	137	10.0	94.6	10.0	10.0	10.0	10.0	11.9	229	2.10*10	327	2.78*10

		Concentration (pg/g d.w)	PFPeS	PFHxS	PFHpS	PFOS	PFNS	PFDS	PFDoDS	HFPO-DA	ADONA	PFECHS	8:2 Cl-PFESA	6:2 Cl-PFESA	∑PFCAs	∑PFSAs	∑PFAS
															^3		^3
Songjiang (n=5)	2007	**Mean**	N/A	10.0	10.0	87.1	N/A	N/A	N/A	N/A	N/A	N/A	N/A	N/A	1.72*10^3	107	1.83*10^3
		**SD**		0	0	39.9									1.13*10^3	39.9	1.16*10^3
		**Median**		10.0	10.0	92.1									1.13*10^3	112	1.20*10^3
	2012	**Mean**	N/A	10.0	10.0	469	N/A	N/A	N/A	N/A	N/A	N/A	N/A	N/A	2.13*10^3	489	2.52*10^3
		**SD**		0	0	267									1.27*10^3	267	1.37*10^3
		**Median**		10.0	10.0	367									3.36*10^3	387	3.59*10^3
	2017	**Mean**	10.0	49.5	10.0	175	10.0	117	10.0	10.0	10.0	10.0	22.5	172	3.06*10^3	382	3.67*10^3
		**SD**	0	36.3	0	85.3	0	58.3	0	0	0	0	10.2	71.8	1.37*10^3	114	1.45*10^3
		**Median**	10.0	29.4	10.0	171	10.0	95.6	10.0	10.0	10.0	10.0	18.8	175	2.77*10^3	359	3.54*10^3
Yangpu	2007	**Mean**	N/A	10.0	10.0	265	N/A	N/A	N/A	N/A	N/A	N/A	N/A	N/A	1.77*10^3	285	2.06*10^3

		Concentration (pg/g d.w)	PFPeS	PFHxS	PFHpS	PFOS	PFNS	PFDS	PFDoDS	HFPO-DA	ADONA	PFECHS	8:2 Cl-PFESA	6:2 Cl-PFESA	∑PFCAs	∑PFSAs	∑PFAS
(n=1)	2012	**Mean**		10.0	10.0	267									714	287	1.00*10^3
	2017	**Mean**	10.0	10.0	10.0	160	10.0	116	10.0	10.0	10.0	10.0	10.0	102	1.98*10^3	326	2.45*10^3

N/A: not analyzed for samples in 2007 and 2012

Table S7. PFAS concentration in abandoned land of Shanghai from 2007 to 2017 (pg/g d.w.).

Compound	2007 (n=12)				2012 (n=14)				2017 (n=5)			
	Min	Max	Mean	Median	Min	Max	Mean	Median	Min	Max	Mean	Median
PFHxA	10.0	292	83.0	53.0	10.0	95.6	30.9	10.0	10.0	148	88.2	91.5
PFHpA	10.0	140	61.7	60.0	10.0	79.9	23.4	10.0	10.0	139	59.9	39.6
PFOA	199	3.06*10^3	1.13*10^3	1.12*10^3	42.5	1.43*10^3	522	315	120	1.99*10^3	997	871
PFNA	38.8	424	159	123	10.0	130	48.2	30.5	10.0	143	71.3	80.6
PFDA	17.0	110	47.4	50.0	10.0	186	49.4	39.7	10.0	163	65.9	54.2
PFUnDA	10.0	137	41.0	33.7	10.0	123	38.0	21.9	10.0	133	55.0	53.7
PFDoDA	10.0	24.2	14.0	12.7	10.0	81.9	17.8	10.0	10.0	37.1	20.5	10.0
PFTriDA	10.0	20.0	11.1	10.0	10.0	44.9	12.5	10.0	10.0	38.6	19.7	10.0
PFPeS	N/A	N/A	N/A	N/A	N/A	N/A	N/A	N/A	10.0	10.0	10.0	10.0
PFHxS	10.0	10.0	10.0	10.0	10.0	10.0	10.0	10.0	10.0	87.2	41.8	35.5
PFHpS	10.0	10.0	10.0	10.0	10.0	10.0	10.0	10.0	10.0	10.0	10.0	10.0
PFOS	25.7	169	78.1	75.0	10.0	455	190	199	21.5	392	135	61.5
PFNS	N/A	N/A	N/A	N/A	N/A	N/A	N/A	N/A	10.0	10.0	10.0	10.0
PFDS	N/A	N/A	N/A	N/A	N/A	N/A	N/A	N/A	40.3	232	128	131
PFDoDS	N/A	N/A	N/A	N/A	N/A	N/A	N/A	N/A	10.0	62.6	20.5	10.0
HFPO-DA	N/A	N/A	N/A	N/A	N/A	N/A	N/A	N/A	10.0	10.0	10.0	10.0

ADONA	N/A	N/A	N/A	N/A	N/A	N/A	N/A	N/A	10.0	10.0	10.0	10.0
PFECHS	N/A	N/A	N/A	N/A	N/A	N/A	N/A	N/A	10.0	10.0	10.0	10.0
8:2 Cl-PFESA	N/A	N/A	N/A	N/A	N/A	N/A	N/A	N/A	10.0	26.9	15.9	10.0
6:2 Cl-PFESA	N/A	N/A	N/A	N/A	N/A	N/A	N/A	N/A	10.0	378	126	109
∑PFCAs	316	3.93*10^3	1.55*10^3	1.50*10^3	113	2.05*10^3	742	442	190	2.79*10^3	1.38*10^3	1.20*10^3
∑PFSAs	45.7	189	98.1	95.0	30.0	475	210	219	207	560	355	268
∑PFAS	367	4.10*10^3	1.65*10^3	1.61*10^3	156	2.26*10^3	952	747	448	3.78*10^3	1.91*10^3	1.63*10^3

N/A: not analyzed for samples in 2007 and 2012

Table S8. PFAS concentration in green-belt soil of Shanghai from 2007 to 2017 (pg/g d.w.).

Compound	2007 (n=9)				2012 (n=7)				2017 (n=22)			
	Min	Max	Mean	Median	Min	Max	Mean	Median	Min	Max	Mean	Median
PFHxA	10.0	360	67.8	10.0	26.7	436	112	60.5	33.5	310	123	106
PFHpA	10.0	181	66.5	47.1	28.6	175	70.2	41.7	30.5	259	97.6	57.6
PFOA	10.0	6.21*10^3	1.39*10^3	1.01*10^3	385	6.94*10^3	1.5*10^3	645	222	7.07*10^3	1.54*10^3	717
PFNA	10.0	417	190	211	10.0	104	45.9	42.4	26.2	214	92.7	75.8
PFDA	10.0	157	52.8	46.4	10.0	81.0	39.0	39.9	10.0	192	68.8	53.4
PFUnDA	10.0	203	47.1	26.0	10.0	83.4	30.4	29.6	10.0	143	63.5	61.5
PFDoDA	10.0	52.4	18.0	11.3	10.0	64.8	27.2	10.0	10.0	72.7	22.8	10.0
PFTrDA	10.0	57.4	15.3	10.0	10.0	33.3	15.3	10.0	10.0	64.2	24.4	16.0
PFPeS	N/A	N/A	N/A	N/A	N/A	N/A	N/A	N/A	10.0	10.0	10.0	10.0
PFHxS	10.0	10.0	10.0	10.0	10.0	10.0	10.0	10.0	10.0	50.7	20.4	10.0
PFHpS	10.0	10.0	10.0	10.0	10.0	35.7	13.7	10.0	10.0	10.0	10.0	10.0
PFOS	10.0	441	132	87.4	73.3	775	298	239	10.0	502	120	82.2
PFNS	N/A	N/A	N/A	N/A	N/A	N/A	N/A	N/A	10.0	10.0	10.0	10.0
PFDS	N/A	N/A	N/A	N/A	N/A	N/A	N/A	N/A	43.5	226	122	111
PFDoDS	N/A	N/A	N/A	N/A	N/A	N/A	N/A	N/A	10.0	60.3	17.4	10.0
HFPO-DA	N/A	N/A	N/A	N/A	N/A	N/A	N/A	N/A	10.0	10.0	10.0	10.0

ADONA	N/A	N/A	N/A	N/A	N/A	N/A	N/A	N/A	10.0	10.0	10.0	10.0
PFECHS	N/A	N/A	N/A	N/A	N/A	N/A	N/A	N/A	10.0	10.0	10.0	10.0
8:2 Cl-PFESA	N/A	N/A	N/A	N/A	N/A	N/A	N/A	N/A	10.0	224	38.9	10.0
6:2 Cl-PFESA	N/A	N/A	N/A	N/A	N/A	N/A	N/A	N/A	10.0	647	160	120
∑PFCAs	80.0	7.51*10^3	1.85*10^3	1.45*10^3	590	7.92*10^3	1.93*10^3	807	432	7.90*10^3	2.03*10^3	1.08*10^3
∑PFSAs	30.0	461	152	107	93.3	820	322	259	175	710	310	292
∑PFAS	110	7.97*10^3	2.00*10^3	1.58*10^3	686	8.74*10^3	2.25*10^3	1.28*10^3	722	8.56*10^3	2.57*10^3	1.58*10^3

N/A: not analyzed for samples in 2007 and 2012

Table S9. PFAS concentration in agricultural soil of Shanghai from 2007 to 2017 (pg/g d.w.).

Compound	2007 (n=30)				2012 (n=29)				2017 (n=15)			
	Min	Max	Mean	Median	Min	Max	Mean	Median	Min	Max	Mean	Median
PFHxA	10.0	144	30.3	10.0	10.0	975	78.7	40.0	55.1	296	149	134
PFHpA	10.0	209	42.3	17.1	10.0	75.9	30.4	26.8	29.8	103	79.4	84.7
PFOA	277	5.00*10^3	738	578	240	1.79*10^3	762	787	572	1.91*10^3	1.23*10^3	1.21*10^3
PFNA	10.0	478	180	147	10.0	236	79.4	70.2	34.1	201	114	95.8
PFDA	10.0	110	43.8	37.9	10.0	206	49.2	39.8	22.0	200	112	110
PFUnDA	10.0	350	44.8	28.2	10.0	192	36.0	25.0	10.0	174	85.4	86.8
PFDoDA	10.0	63.2	14.0	10.0	10.0	101	20.3	10.0	10.0	89.1	30.0	28.5
PFTrDA	10.0	25.3	11.7	10.0	10.0	34.7	10.9	10.0	10.0	51.5	29.4	33.3
PFPeS	N/A	N/A	N/A	N/A	N/A	N/A	N/A	N/A	10.0	10.0	10.0	10.0
PFHxS	10.0	10.0	10.0	10.0	10.0	50.6	11.4	10.0	10.0	108	47.9	41.5
PFHpS	10.0	10.0	10.0	10.0	10.0	135	14.7	10.0	10.0	10.0	10.0	10.0
PFOS	15.3	190	85.6	79.4	32.4	1.99*10^3	252	161	44.6	1.04*10^3	205	174
PFNS	N/A	N/A	N/A	N/A	N/A	N/A	N/A	N/A	10.0	10.0	10.0	10.0
PFDS	N/A	N/A	N/A	N/A	N/A	N/A	N/A	N/A	82.6	313	164	170
PFDoDS	N/A	N/A	N/A	N/A	N/A	N/A	N/A	N/A	10.0	110	20.7	10.0
HFPO-DA	N/A	N/A	N/A	N/A	N/A	N/A	N/A	N/A	10.0	10.0	10.0	10.0

Compound	2007 (n=30)				2012 (n=29)				2017 (n=15)			
	Min	Max	Mean	Median	Min	Max	Mean	Median	Min	Max	Mean	Median
ADONA	N/A	N/A	N/A	N/A	N/A	N/A	N/A	N/A	10.0	10.0	10.0	10.0
PFECHS	N/A	N/A	N/A	N/A	N/A	N/A	N/A	N/A	10.0	10.0	10.0	10.0
8:2 Cl-PFESA	N/A	N/A	N/A	N/A	N/A	N/A	N/A	N/A	10.0	220	34.3	14.5
6:2 Cl-PFESA	N/A	N/A	N/A	N/A	N/A	N/A	N/A	N/A	10.0	1.68*10^3	374	177
∑PFCAs	440	5.62*10^3	1.10*10^3	912	315	3.36*10^3	1.07*10^3	1.02*10^3	746	2.80*10^3	1.83*10^3	1.82*10^3
∑PFSAs	35.3	259	106	99.4	62.0	2.01*10^3	278	181	215	1.17*10^3	467	351
∑PFAS	540	5.88*10^3	1.21*10^3	991	432	4.08*10^3	1.34*10^3	1.22*10^3	1.16*10^3	4.20*10^3	2.73*10^3	2.97*10^3

N/A: not analyzed for samples in 2007 and 2012

Table S10. PFAS concentration in forest land of Shanghai from 2007 to 2017 (pg/g d.w.).

Compound	2007 (n=3)				2012 (n=4)				2017 (n=12)			
	Min	Max	Mean	Median	Min	Max	Mean	Median	Min	Max	Mean	Median
PFHxA	10.0	44.0	21.3	10.0	29.6	101	72.9	80.3	85.7	1.04e3	239	160
PFHpA	10.0	155	60.7	17.6	10.0	221	112	108	51.3	220	129	119
PFOA	572	3.92*10^3	1.79*10^3	886	579	1.10*10^4	4.17*10^3	2.55*10^3	984	6.96*10^3	2.06*10^3	1.55*10^3
PFNA	112	1.72*10^3	858	740	10.0	275	119	95.3	45.5	206	114	118
PFDA	51.3	243	176	235	25.2	398	145	77.1	27.5	218	109	98.6
PFUnDA	27.4	285	159	166	10.0	275	106	70.0	26.8	170	92.0	80.9
PFDoDA	13.4	178	121	173	10.0	164	65.3	43.6	10.0	80.2	32.2	27.7
PFTrDA	12.6	77.1	44.6	44.1	10.0	35.9	16.5	10.0	10.0	89.6	36.8	31.2
PFPeS	N/A	N/A	N/A	N/A	N/A	N/A	N/A	N/A	10.0	10.0	10.0	10.0
PFHxS	10.0	10.0	10.0	10.0	10.0	10.0	10.0	10.0	10.0	120	37.9	27.6
PFHpS	10.0	10.0	10.0	10.0	10.0	10.0	10.0	10.0	10.0	10.0	10.0	10.0
PFOS	42.5	1.51*10^3	676	476	32.1	862	313	179	42.4	6.02*10^3	601	102
PFNS	N/A	N/A	N/A	N/A	N/A	N/A	N/A	N/A	10.0	10.0	10.0	10.0
PFDS	N/A	N/A	N/A	N/A	N/A	N/A	N/A	N/A	70.8	177	114	105
PFDoDS	N/A	N/A	N/A	N/A	N/A	N/A	N/A	N/A	10.0	43.0	12.7	10.0
HFPO-DA	N/A	N/A	N/A	N/A	N/A	N/A	N/A	N/A	10.0	10.0	10.0	10.0

Compound	2007 (n=3)				2012 (n=4)				2017 (n=12)			
	Min	Max	Mean	Median	Min	Max	Mean	Median	Min	Max	Mean	Median
ADONA	N/A	N/A	N/A	N/A	N/A	N/A	N/A	N/A	10.0	10.0	10.0	10.0
PFECHS	N/A	N/A	N/A	N/A	N/A	N/A	N/A	N/A	10.0	10.0	10.0	10.0
8:2 Cl-PFESA	N/A	N/A	N/A	N/A	N/A	N/A	N/A	N/A	10.0	469	65.9	13.8
6:2 Cl-PFESA	N/A	N/A	N/A	N/A	N/A	N/A	N/A	N/A	77.4	8.67*10^3	1.30*10^3	174
∑PFCAs	850	6.59*10^3	3.23*10^3	2.26*10^3	683	1.24*10^4	4.80*10^3	3.04*10^3	1.33*10^3	8.46*10^3	2.81*10^3	2.21*10^3
∑PFSAs	62.5	1.53*10^3	696	496	52.1	882	333	199	188	6.33*10^3	795	307
∑PFAS	912	8.12*10^3	3.93*10^3	2.76*10^3	735	1.33*10^4	5.14*10^3	3.24*10^3	2.09*10^3	1.83*10^4	5.00*10^3	3.18*10^3

N/A: not analyzed for samples in 2007 and 2012

Table S11. PFAS concentration (expressed in F-equivalent) (ng F/g), EOF concentration (ng F/g), and mass balance analysis of EOF (%) in soil samples from Shanghai.

	2007				2012				2017			
	Min	Max	Mean	Median	Min	Max	Mean	Median	Min	Max	Mean	Median
EOF	n.d.	60.2	23.8	17.3	n.d.	32.8	20.1	17.0	n.d.	57.8	24.2	24.8
PFAS	0.210	6.15	1.13	0.760	0.0932	9.17	1.11	0.805	0.306	11.3	2.08	1.63
PFAS/EOF	1.68	48.6	9.92	5.47	2.38	32.5	7.29	4.87	1.10	75.9	11.8	9.85

n.d.: <MDL

Table S12. PFAS concentration (expressed in F-equivalent) (ng F/g), EOF concentration (ng F/g), and mass balance analysis of EOF (%) in soil samples from various areas of Shanghai.

		2007				2012				2017			
		Min	Max	Mean	Median	Min	Max	Mean	Median	Min	Max	Mean	Median
Baoshan (n=2)	EOF	18.4	18.4	18.4	18.4	32.8	32.8	32.8	32.8	25.5	35.9	30.7	30.7
	PFAS	0.671	1.10	0.883	0.883	0.232	0.879	0.555	0.555	0.884	1.55	1.22	1.22
	PFAS/EOF	5.95	5.95	5.95	5.95	2.68	2.68	2.68	2.68	3.47	4.33	3.90	3.90
Chongming Island (n=4)	EOF	13.1	22.6	17.8	17.8	13.5	16.8	15.2	15.2	n.d.	n.d.		
	PFAS	0.462	0.749	0.617	0.628	0.311	0.489	0.398	0.397	0.781	1.079	0.964	0.998
	PFAS/EOF	3.32	4.58	3.95	3.95	2.58	2.66	2.62	2.62	n.d.	n.d.		
Fengxian (n=4)	EOF	n.d.	n.d.			n.d.	n.d.			14.0	14.0	14.0	14.0
	PFAS	0.367	1.10	0.692	0.651	0.341	1.25	0.790	0.785	1.00	2.63	1.55	1.28
	PFAS/EOF	n.d.	n.d.			n.d.	n.d.			18.9	18.9	18.9	18.9
Jiading (n=6)	EOF	15.8	15.8	15.8	15.8	17.2	18.5	17.9	17.9	22.8	57.8	34.3	31.0
	PFAS	0.284	1.40	0.734	0.556	0.330	1.54	0.618	0.452	0.530	3.18	1.62	1.33
	PFAS/EOF	8.84	8.84	8.84	8.84	2.89	8.91	5.90	5.90	1.10	10.9	5.22	4.54
Jinshan (n=7)	EOF	13.4	60.2	37.6	39.2	14.5	30.1	21.9	21.5	13.5	25.0	17.9	17.3
	PFAS	0.307	1.01	0.658	0.754	0.731	1.34	0.969	0.961	2.02	11.3	3.88	2.55
	PFAS/EOF	1.68	2.29	1.97	1.94	2.68	7.35	4.94	4.87	10.0	75.9	27.5	15.0
Minhang (n=6)	EOF	12.7	27.3	20.0	20.0	15.2	15.2	15.2	15.2	16.0	32.1	24.3	24.8
	PFAS	0.451	6.15	2.92	2.39	0.440	5.98	1.80	0.863	0.306	5.92	2.16	1.11
	PFAS/EOF	20.6	48.6	34.6	34.6	5.42	5.42	5.42	5.42	4.86	11.7	7.44	5.75
Pudong New Area (n=13)	EOF	15.0	15.0	15.0	15.0	18.9	18.9	18.9	18.9	12.9	30.6	21.6	21.4
	PFAS	0.265	2.26	0.972	0.660	0.093	2.11	0.895	1.08	0.602	6.24	1.90	1.50
	PFAS/EOF	7.46	7.46	7.46	7.46	5.72	5.72	5.72	5.72	3.01	25.8	11.1	11.7

		2007				2012				2017			
		Min	Max	Mean	Median	Min	Max	Mean	Median	Min	Max	Mean	Median
Qingpu (n=6)	EOF	16.2	31.4	23.8	23.8	16.5	16.5	16.5	16.5	19.5	26.4	22.7	22.5
	PFAS	0.210	1.57	1.07	1.08	0.138	1.1058917	0.648	0.673	1.10	2.57	1.85	1.94
	PFAS/EOF	5.00	8.83	6.91	6.91	2.38	2.38	2.38	2.38	7.42	10.16	9.37	9.96
Songjiang (n=5)	EOF	n.d.	n.d.			16.7	28.2	22.4	22.4	15.3	15.3	15.3	15.3
	PFAS	0.651	2.87	1.26	0.816	0.468	9.17	3.20	2.47	1.42	4.36	2.52	2.44
	PFAS/EOF	n.d.	n.d.			16.6	32.5	24.5	24.5	16.1	16.1	16.1	16.1
Yangpu (n=1)	EOF	n.d.	n.d.			n.d.	n.d.			26.8	26.8	26.8	26.8
	PFAS	1.47	1.47	1.47	1.47	0.660	0.660	0.660	0.660	1.67	1.67	1.67	1.67
	PFAS/EOF	n.d.	n.d.			n.d.	n.d.			6.23	6.23	6.23	6.23

n.d.: <MDL

Table S13. PFAS concentration (expressed in F equivalent) (ng-F/g), EOF concentration (ng F/g), and mass balance analysis of EOF (%) in different soil use from Shanghai.

		2007			2012			2017		
		EOF	PFAS	PFAS/EOF	EOF	PFAS	PFAS/EOF	EOF	PFAS	PFAS/EOF
Abandoned soil	Min	n.d.	0.210	n.d.	n.d.	0.0932	n.d.	n.d.	0.306	n.d.
	Max	16.2	2.87	8.84	17.2	1.54	8.91	25.3	2.57	10.2
	Mean	16.0	1.13	8.83	16.9	0.628	5.65	25.3	1.30	10.2
	Median	16.0	1.11	8.83	16.9	0.518	5.65	25.3	1.11	10.2
Agricultural soil	Min	n.d.	0.307	n.d.	n.d.	0.232	n.d.	n.d.	0.788	n.d.
	Max	60.2	4.14	5.00	30.1	2.76	16.6	32.8	2.76	15.0
	Mean	30.0	0.826	3.13	18.7	0.889	5.56	23.2	1.84	10.8
	Median	27.0	0.665	2.80	16.7	0.808	4.87	22.4	1.97	10.1
Forest land	Min	n.d.	0.612	n.d.	n.d.	0.440	n.d.	n.d.	1.42	n.d.
	Max	12.7	6.15	48.6	28.2	9.17	32.5	30.6	11.3	75.9
	Mean	12.7	2.94	48.6	28.2	3.51	32.5	19.2	3.29	21.3
	Median	12.7	2.07	48.6	28.2	2.22	32.5	15.5	2.20	14.1
Green-belt soil	Min	n.d.	0.451	n.d.	n.d.	0.489	n.d.	n.d.	0.530	n.d.
	Max	27.3	5.63	20.6	32.8	5.98	2.68	57.8	5.92	21.7
	Mean	20.2	1.56	11.3	32.8	1.52	2.68	27.5	1.76	6.65
	Median	18.4	1.11	7.46	32.8	0.856	2.68	26.8	1.07	4.34

n.d.: <MDL

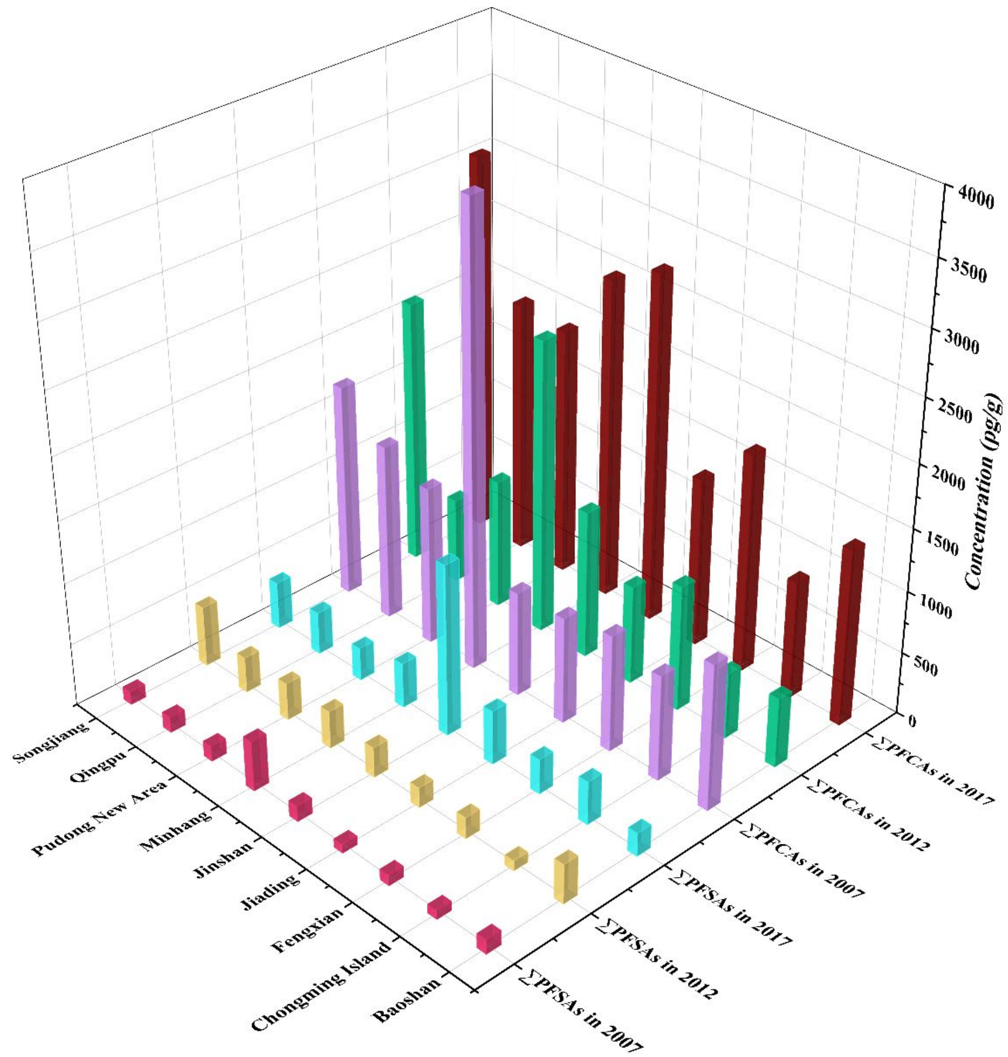

Figure S1. The concentration of PFCAs and PFSAs in soils from Shanghai.

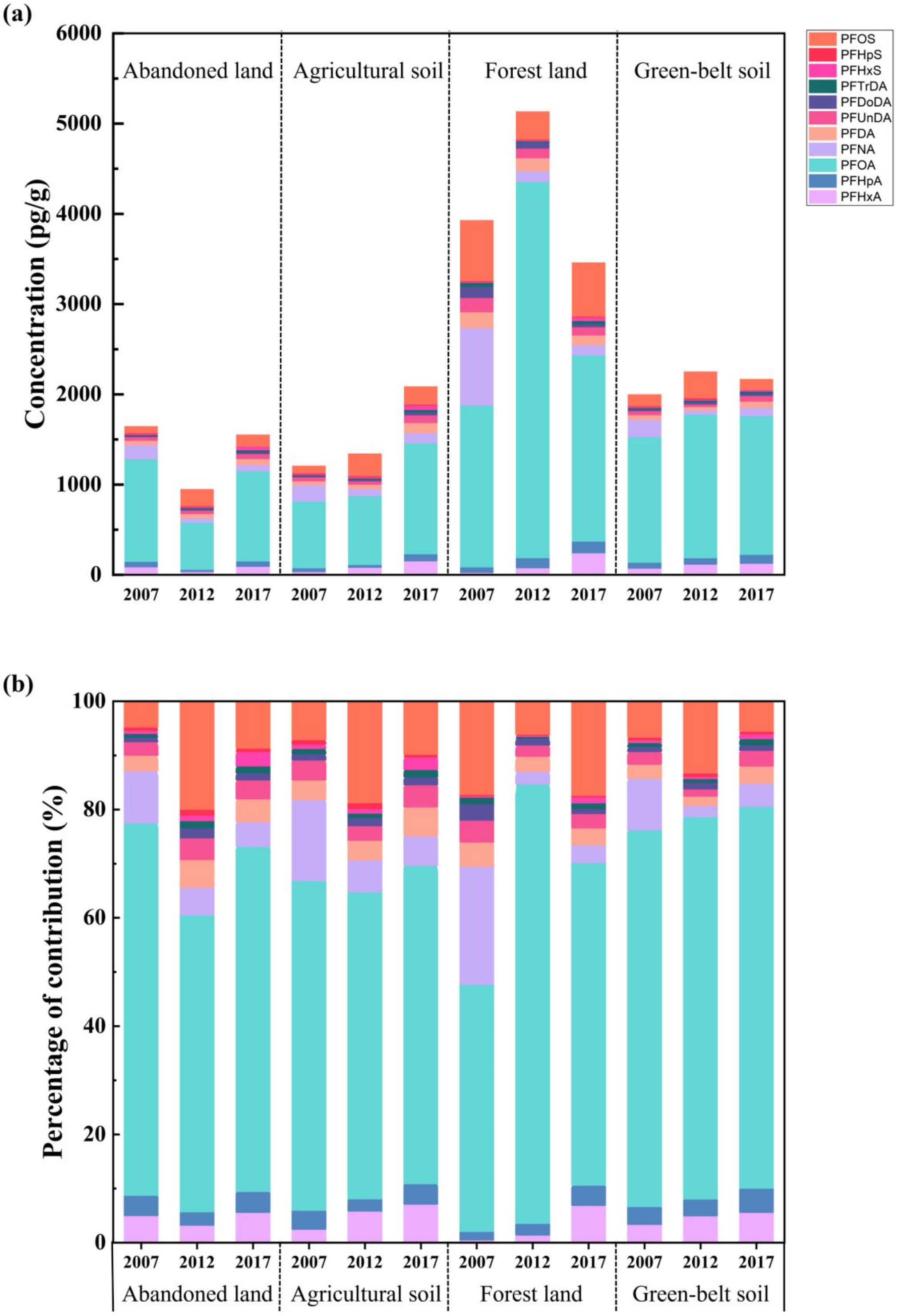

Figure S2. The 11 PFAS concentrations (a) and compositional profiles (b) of different land uses in Shanghai

References

(1) Kim, E. J.; Park, Y.-M.; Park, J.-E.; Kim, J.-g. Distributions of new Stockholm Convention POPs in soils across South Korea. *Science of The Total Environment* **2014**, *476-477*, 327-335.

(2) Tan, B.; Wang, T.; Wang, P.; Luo, W.; Lu, Y.; Romesh, K. Y.; Giesy, J. P. Perfluoroalkyl substances in soils around the Nepali Koshi River: levels, distribution, and mass balance. *Environmental Science and Pollution Research* **2014**, *21* (15), 9201-9211.

(3) Li, F.; Zhang, C.; Qu, Y.; Chen, J.; Chen, L.; Liu, Y.; Zhou, Q. Quantitative characterization of short- and long-chain perfluorinated acids in solid matrices in Shanghai, China. *Science of The Total Environment* **2010**, *408* (3), 617-623.

(4) Wang, Q.; Zhao, Z.; Ruan, Y.; Li, J.; Sun, H.; Zhang, G. Occurrence and distribution of perfluorooctanoic acid (PFOA) and perfluorooctanesulfonic acid (PFOS) in natural forest soils: A nationwide study in China. *Science of The Total Environment* **2018**, *645*, 596-602.

(5) Lee, Y.-M.; Lee, J.-Y.; Kim, M.-K.; Yang, H.; Lee, J.-E.; Son, Y.; Kho, Y.; Choi, K.; Zoh, K.-D. Concentration and distribution of per- and polyfluoroalkyl substances (PFAS) in the Asan Lake area of South Korea. *Journal of Hazardous Materials* **2020**, *381*.
